# Thiol–Disulfide Exchange Coordinates the Release of Nitric Oxide and Dexamethasone for Synergistic Regulation of Intestinal Microenvironment in Colitis

**DOI:** 10.34133/research.0204

**Published:** 2023-08-01

**Authors:** Junna Lu, Tongfei Shi, Chengxin Shi, Fangman Chen, Chao Yang, Xiaochun Xie, Zheng Wang, He Shen, Jiaqi Xu, Kam W. Leong, Dan Shao

**Affiliations:** ^1^ School of Biomedical Sciences and Engineering, South China University of Technology, Guangzhou International Campus, Guangzhou, Guangdong 510006, China.; ^2^ National Engineering Research Center for Tissue Restoration and Reconstruction, South China University of Technology, Guangdong 510006, China.; ^3^ Department of Plastic and Aesthetic Center, The First Affiliated Hospital of Zhejiang University, Hangzhou 310000, China.; ^4^ Department of Biomedical Engineering, Columbia University, New York, NY 10027, USA.; ^5^ School of Medicine, South China University of Technology, Guangzhou, Guangdong 510006, China.; ^6^ CAS Key Laboratory of Nano-Bio Interface, Suzhou Institute of Nano-Tech and NanoBionics, Chinese Academy of Sciences, Suzhou 215123, China.; ^7^ CAS Key Laboratory for Biomedical Effects of Nanomaterials & Nanosafety, CAS Center for Excellence in Nanoscience, National Center for Nanoscience and Technology, Beijing 100190, China.; ^8^ Guangdong Provincial Key Laboratory of Biomedical Engineering, South China University of Technology, Guangzhou 510006, China.; ^9^ Key Laboratory of Biomedical Materials and Engineering of the Ministry of Education, South China University of Technology, Guangzhou 510006, China.

## Abstract

The cell-specific functions of nitric oxide (NO) in the intestinal microenvironment orchestrate its therapeutic effects in ulcerative colitis. While most biomaterials show promise by eliciting the characteristics of NO, the insufficient storage, burst release, and pro-inflammatory side effects of NO remain as challenges. Herein, we report the development of thiol–disulfide hybrid mesoporous organosilica nanoparticles (MONs) that improve the storage and sustained release of NO, broadening the therapeutic window of NO-based therapy against colitis. The tailored NO-storing nanomaterials coordinated the release of NO and the immunoregulator dexamethasone (Dex) in the intestinal microenvironment, specifically integrating the alleviation of oxidative stress in enterocytes and the reversal of NO-exacerbated macrophage activation. Mechanistically, such a synchronous operation was achieved by a self-motivated process wherein the thiyl radicals produced by NO release cleaved the disulfide bonds to degrade the matrix and release Dex via thiol–disulfide exchange. Specifically, the MON-mediated combination of NO and Dex greatly ameliorated intractable colitis compared with 5-aminosalicylic acid, even after delayed treatment. Together, our results reveal a key contribution of synergistic modulation of the intestinal microenvironment in NO-based colitis therapy and introduce thiol–disulfide hybrid nanotherapeutics for the management of inflammatory diseases and cancer.

## Introduction

Ulcerative colitis (UC) is a major subcategory of inflammatory bowel disease characterized by chronic intestinal inflammation that follows a pattern of recurrence and remission [1,2]. In clinical settings, the long-term use of traditional immunoregulatory drugs, including 5-aminosalicylic acid (5-SA) [3], corticosteroids [4], and anti-tumor necrosis factor-α (TNF-α) agents [5,6], may cause systemic side effects and serious complications [7]. Nitric oxide (NO), the first endogenous gasotransmitter to be identified, markedly affects various physiological and pathological processes [8], such as vasodilation, the host immune response, and tissue repair [9,10]. Given its important role in regulating gastrointestinal integrity [11], NO has become a promising immunoregulatory agent for colitis treatment. Indeed, in dextran sulfate sodium (DSS)-induced colitis [12,13], a moderate dose of NO provides benefits against tissue injury, whereas a high dose of NO exacerbates intestinal inflammation [14–17]. In such cases, enterocyte-derived NO alleviates colitis by decreasing tissue damage, whereas NO produced from immune cells is associated with macrophage activation and inflammation [16]. These paradoxical and opposing findings hinder the implementation of NO-related treatments for colitis [14,16,18–21]. Therefore, there is a crucial need to modulate the cell-specific manner of NO release in the intestinal microenvironment. With these findings in mind, we hypothesize that a combination strategy that not only maintains the protective effect of NO in enterocytes but also reverses its side effects in intestinal macrophages will be beneficial for NO-based colitis therapy.

To address this unmet need, it is essential to codeliver NO and immunoregulators to the inflamed intestine. Currently, continuous efforts have been made to fabricate NO-storing donors [22] or materials [23,24] that can elicit the characteristics of NO. As an endogenous class of NO donors [25–27], S-nitrosothiols (SNOs) can be synthesized under simple and mild conditions [28]. To overcome their low NO storage per donor molecule, the integration of SNO precursors and nanoparticulate materials enables improved NO storage per delivery carrier but does not fully address the uncontrollable release of NO. In particular, the codelivery of NO and drugs has been achieved with thiol-functionalized materials, including polymer-, inorganic-, and metal-based nanoparticles, to treat various diseases [29–32]. Despite these advances, burst NO release and sustained drug release are unable to occur in a synchronous manner, resulting in insufficient outcomes of combination therapy [32–34]. These characteristics are major challenges for the development of on-demand codelivery systems that can coordinate the release of NO and immunoregulators for synergistic colitis therapy with reduced side effects.

**Fig. 1.**
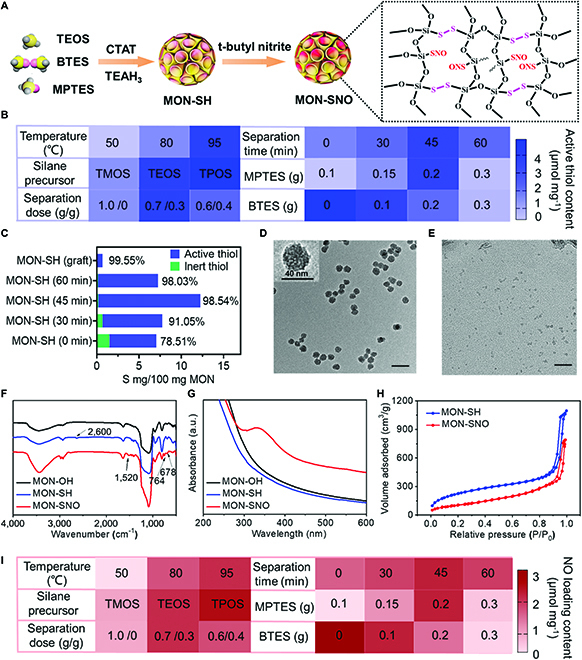
Fabrication and characterization of the thiol–disulfide hybrid MON with high NO loading contents. (A) Schematic diagram for the preparation of the MON-SNO. (B) Quantitative analysis of active thiol group contents (μmol mg^−1^) in MON-SH by Ellman’s reagent. (C) The contents of active and inert thiol group in MON-SH by ICP-MS and Ellman’s reagent. (D) TEM images of MON-SNO. Scale bar: 200 μm. (E) TEM images of MON-SNO were incubated in SBF for 48 h. Scale bar: 200 μm. (F to H) FTIR spectra (F), UV–Vis absorption spectra (G), and N_2_ adsorption–desorption isotherm (H) of MON. (I) Quantitative analysis of NO contents (μmol mg^−1^) in MON-SNO.

Herein, we specifically created an organic–inorganic hybrid nanoplatform for the synchronous release of NO and an immunoregulator that efficiently prevented and treated colitis by synergistically regulating the intestinal microenvironment. As illustrated in Fig. 1A, we introduced both thiol and disulfide moieties into the framework of silica to fabricate thiol–disulfide hybrid mesoporous organosilica nanoparticles (MONs). After transforming the thiol group to SNO through a nitrosation process, the NO-storing nanomaterials encapsulated the immunoregulator dexamethasone (Dex) [35] to form MON-SNO@Dex. Compared with surface-grafted MON-SNO, the framework-doped MON-SNO exhibited 10-fold higher NO storage and dramatically slower burst NO release, further broadening the therapeutic window of NO-based therapy against colitis. On the basis of these findings, we optimized the MON to synchronously release NO and Dex for synergistic regulation of the intestinal microenvironment in colitis, in which sustainably released NO alleviated oxidative stress in enterocytes, while sufficient Dex inhibited the NO-exacerbated M1 polarization of macrophages. Importantly, we deciphered the prominent role that thiol–disulfide exchange plays in this process, as the NO released from the SNO group produces thiyl radicals, which cleave disulfide bonds to trigger matrix degradation and facilitate the release of Dex from the hybrid silica framework. Together, our findings suggest a thiol–disulfide hybrid nanoplatform that can synchronously release NO and an immunoregulator, shedding light on the development of safe and potentially effective NO-based regimens for the treatment of colitis and other inflammatory diseases.

## Results

### Synthesis and optimization of thiol–disulfide hybrid MON with high NO contents and matrix-degrading effects

We prepared a library of thiol–disulfide hybrid MON through hydrolysis and the co-condensation of different silane precursors (Fig. 1A and Fig. S1). After investigating the effects produced by different types of inorganosilanes, the precursor separation mode, and temperature on nanoparticle formation, we found that the content of active thiol groups in the hybrid MON could be tuned by specifically adjusting the ratios of tetraethylorthosilicate (TEOS), 3-mercaptopropyltriethoxysilane (MPTES), and bis(triethoxysilylpropyl) disulfide (BTES) (Fig. 1B). The developed MONs were uniform and exhibited a similar particle size (40 to 50 nm), mesoporous pore size (2 to 4 nm), and negative charge (−15 to −25 mV) (additional characterization details can be found in Figs. S1 to S4 and Tables S1 and S2). Importantly, increasing the amount of the thiol precursor, decreasing the content of the disulfide precursor, and prolonging post-addition time together increased the active thiol content in the MON (Fig. 1B). Given that the content and distribution of active thiol groups affect NO loading and matrix degradation, we explored the distribution of active thiol groups in the MON-SH framework by inductively coupled plasma mass spectrometry (ICP-MS) and Ellman’s reagent. It was found that the inert thiol content decreased when the introduction of the organosilica precursor was delayed (Fig. 1C and Fig. S5).

Next, we nitrosated the thiol group to generate the SNO group through a nitrite intermediate and optimized the best hybrid MON with the highest thiol content (3.65 μmol mg^−1^), which exhibited considerable matrix degradation after 48 h of incubation (Fig. 1D and E). The Fourier transform infrared (FTIR) spectra of MON-SH confirmed the presence of thiol groups (2,600 cm^−1^) in the organosilica framework, while 2 new characteristic peaks of -S-N= and -N=O- at 764 and 1,520 cm^−1^, respectively, were found in MON-SNO (Fig. 1F). Moreover, the ultraviolet–visible (UV–Vis) spectrum displayed the characteristic absorption of the SNO group in the range of 330 to 360 nm (Fig. 1G). As expected, hybrid MON with a higher active thiol content in the framework can contain more NO (Fig. 1I), up to 2.26 μmol mg^−1^ (more than 10-fold higher than other NO-storing materials, which is summarized in Table S3). In addition, the nitrogen adsorption–desorption plots of MON-SNO exhibited type IV isotherms (Fig. 1H) [36]. The Brunauer–Emmett–Teller surface area, total pore volume, and average pore size of the MON-SNO were determined to be 384.41 m^2^ g^−1^, 1.18 cm^3^ g^−1^, and 2.4 nm, respectively. Taken together, we successfully tailored a series of thiol–disulfide hybrid MON with a similar particle size but with different thiol contents and degradation behaviors, and finally optimized the best MON to integrate the high NO loading and considerable biodegradability.

### Framework-doped MON-SNO sustained the release of NO to alleviate oxidative damage to enterocytes and broaden the therapeutic window for colitis

**Fig. 2.**
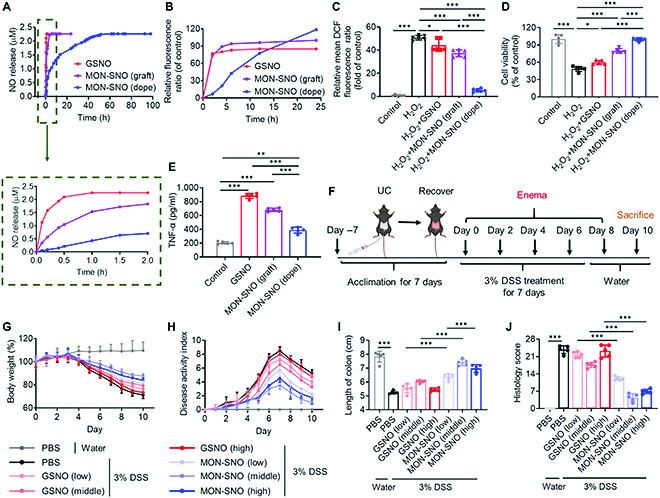
Framework-doped MON-SNO possessed sustained NO release for alleviating oxidative damage of enterocytes and broadened the therapeutic window of colitis. (A) Cumulative NO release profiles of GSNO, MON-SNO (graft), and MON-SNO (dope) in PBS solution. (B) Cumulative NO release profiles of GSNO, MON-SNO (graft), and MON-SNO (dope) in HIEC-6 cells. (C and D) Relative fluorescence intensity of oxidized DCF in HIEC-6 cells (C) and the viability of HIEC-6 cells (D) after incubation with different NO-storing formulations in the presence of H_2_O_2_ (100 μM) (*n* = 5). (E) RAW 264.7 macrophages were stimulated with GSNO, MON-SNO (graft), and MON-SNO (dope). Supernatants were assayed for TNF-α by ELISA (*n* = 5). (F) C57BL/6 mice were provided with 3% DSS for 7 days. On days 0, 2, 4, 6, and 8, rectal enema of PBS, GSNO, and MON-SNO (dope) at the same dosage of NO (1.13, 11.3, and 33.9 μmol kg^−1^ for low, middle, and high doses, respectively). (G and H) Daily body weight (G) and DAI changes (H) of mice in each group for 10 days. (I and J) On day 10, mice were euthanized and colon length (I) and colonic damage scores (J) were measured (*n* = 5). Data were presented as mean ± SD and the statistical significance was calculated via one-way ANOVA with Tukey’s multiple comparisons test. *P < 0.05, **P < 0.01, ***P < 0.001.

Given that burst release of NO marked hampers its function under various physiological and pathological conditions, sustained NO release is highly appropriate to achieve therapeutic goals [28]. To demonstrate the advantages of the framework doping strategy to introduce thiol groups, we prepared thiol-functionalized MON through a traditional surface grafting strategy in parallel for comparison, which exhibited 10-fold lower thiol content (0.184 vs. 3.65 μmol mg^−1^) and NO storage (0.15 vs. 2.26 μmol mg^−1^). As shown in Fig. 2A, framework-doped MON-SNO exhibited sustained NO release (37 °C, pH 7.4, 100 rpm), and approximately 30%, 75%, and almost all of the NO were released at 2, 24, and 72 h, respectively. In contrast, more than 80% of the NO was abruptly released from the surface-grafted MON-SNO within 2 h. Moreover, complete NO release was observed over 1 h from S-nitrosoglutathione (GSNO), a classic NO donor [37]. It is worth noting that all of the thiol-doped MON exhibited similar NO release behavior regardless of disulfide bond inclusion (Fig. S6), indicating that thiol groups that are well-distributed in the silica framework will improve the stability of SNO. The release kinetics of NO under different release conditions (temperature, pH, and shaking speed) also exhibited prolonged release (Fig. S7).

We then compared the intracellular NO level after its release from GSNO, surface-grafted MON-SNO, and framework-doped MON-SNO after incubation with HIEC-6 enterocytes and RAW 264.7 macrophages. The temporal profiles of NO levels were evaluated by confocal laser scanning microscopy (CLSM) using 3-amino,4-aminomethyl-2′,7′-difluorescein, diacetate (DAF-FM DA), a reagent used to visualize NO [38]. The NO signal was markedly increased with prolonged incubation with the framework-doped MON-SNO (Fig. 2B and Fig. S8). However, after incubation with GSNO and the surface-grafted MON-SNO, stronger fluorescence intensity was exhibited at early time points (2 h) (Fig. 2B and Fig. S8), consistent with their NO burst release. After determining the NO release profile, we investigated the cytotoxicity of GSNO and 2 types of MON-SNO by the sulforhodamine assay [39] with the same NO concentration. The framework-doped MON-SNO exhibited less toxicity than other NO-storing molecules/materials even at high concentrations (>500 μM NO) (Fig. S9). Since oxidative stress is considered a crucial microenvironmental factor involved in intestinal damage [40], we explored whether NO-storing MON could protect enterocytes from oxidative damage. As shown in Fig. 2C and D and Fig. S10, H_2_O_2_ pretreatment not only evoked remarkable reactive oxygen species (ROS) production but also induced marked HIEC-6 cell death. However, the framework-doped MON-SNO markedly promoted ROS scavenging and cytoprotection in H_2_O_2_-challenged enterocytes compared with both GSNO and surface-grafted MON-SNO. It is worth noting that all 3 types of NO-storing molecules/materials markedly boosted pro-inflammatory cytokine TNF-α, interleukin-1β (IL-1β), and interleukin-6 (IL-6) secretion in macrophages. However, the framework-doped MON-SNO evoked the least NO-exacerbated macrophage activation due to its sustained NO release (Fig. 2E and Fig. S11). Collectively, these data show that framework-doped MON-SNO sustainably released NO, which greatly alleviated oxidative stress and enterocyte damage while inducing less macrophage activation.

To test the hypothesis that the dose of NO affects the therapeutic outcome in the treatment of colitis, we selected 3 different concentrations of GSNO and framework-doped MON-SNO with the same dosages of NO (1.13, 11.3, and 33.9 μmol kg^−1^ for the low, middle, and high doses, respectively). For the colitis prevention experiment, mice were fed 3% DSS (w/v) for 7 consecutive days to induce acute colitis [41] (Fig. 2F and Fig. S12). The mice received rectal enemas [42] of GSNO or MON-SNO with the aid of the poloxamer 407 (Pluronic F127) hydrogel [43] every other day. As shown in Fig. 2G to J and Fig. S12, the middle doses of MON-SNO and GSNO displayed marginally better results than the low and high doses in terms of body weight loss reversal, increased disease activity index (DAI), colon shortening, and colonic tissue damage. Therefore, MON-SNO possesses a greater protective effect than GSNO, especially at high doses, indicating that sustained NO release may reduce the side effects of NO. These results allowed us to conclude that framework-doped MON-SNO broadened the therapeutic window of NO for the treatment of colitis with fewer side effects.

### Framework-doped MON-SNO synchronously released NO and Dex through thiol–disulfide exchange

**Fig. 3.**
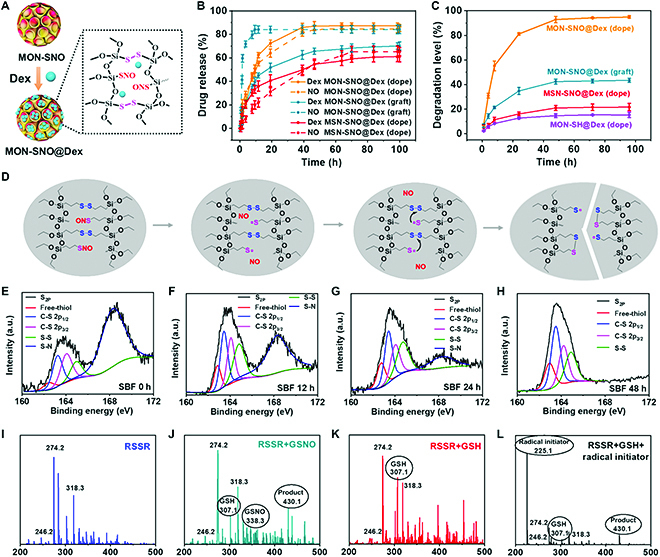
Framework-doped MON-SNO synchronously released NO and Dex through a thiol–disulfide exchange. (A) Schematic diagram for the preparation of the MON-SNO@Dex. (B) Release profiles of NO and Dex from MON-SNO@Dex (dope), MON-SNO@Dex (graft), and MSN-SNO@Dex (dope). (C) The degradation level (the content of Si by ICP-MS) of MON-SNO@Dex (dope), MON-SNO@Dex (graft), MSN-SNO@Dex (dope), and MON-SH@Dex (dope) in SBF. (D) The degradation mechanism of MON-SNO (dope) via thiol–disulfide exchange reaction. (E to H) S_2P_ of MON-SNO@Dex (dope) after incubation in SBF for 0 h (E), 12 h (F), 24 h (G), and 48 h (H). Free thiol (162.5 eV); C-S 2p_1/2_ (163.7 eV); C-S 2p_3/2_ (164.3 eV); S-S (165 eV); S-N (168.5 eV) regions. (I) ESI-MS of RSSR. (J) ESI-MS of the reaction product of RSSR (10 mM) and GSNO (2 mM) for 6 h. (K) ESI-MS of the reaction product of RSSR (10 mM) and GSH (2 mM) for 6 h. (L) ESI-MS of the reaction product of RSSR (10 mM), GSH (2 mM), and radical initiator (2,2-dimethoxy-2-phenylacetophenone, 30 mM) for 6 h. Data were presented as mean ± SD.

Coordinating the release of NO and immunoregulators is crucial to protect against enterocyte damage and synergizes with the inhibition of macrophage activation, leading to efficient and safe colitis therapy. Given that corticosteroids are the mainstay for the treatment of moderate to severe colitis [44], Dex [45,46] was selected as the model drug for combination treatment in this study. Framework-doped MON-SNO possessed a high loading capacity (25.37% ± 2.46%) of Dex (Fig. 3A and Fig. S13). Additionally, framework-doped MON-SNO without disulfide bonds (named MSN-SNO) were selected as a control to determine how disulfide bonds participate in matrix degradation and drug release. The in vitro drug release profiles for 3 types of Dex-loaded nanomaterials are illustrated in Fig. 3B. Framework-doped MON-SNO@Dex and MSN-SNO@Dex expectedly exhibited prolonged NO release compared with surface-grafted MON-SNO@Dex. Importantly, the framework-doped MON-SNO@Dex exhibited synchronous release of NO and Dex that was more coordinated than the release from the surface-grafted MON-SNO@Dex and framework-doped MSN-SNO@Dex. Interestingly, the release of Dex from MON-SH@Dex was relatively slower (Fig. S14), implying that NO release might affect the process of matrix degradation and subsequent Dex release.

To decipher whether NO release facilitates Dex release, we first determined the degradation of the matrix during the NO release process. We found that MSN without disulfide incorporation exhibited slow degradation behavior in simulated body fluid (SBF) (Fig. 3C and Fig. S15). Importantly, SNO modification markedly improved the degradation of framework-doped MON. In particular, framework-doped MON with greater thiol–disulfide incorporation in the framework exhibited a faster degradation rate than surface-grafted MON with fewer thiol–disulfide interactions [47,48]. Given that thiyl radicals can facilitate thiol–disulfide exchange [49], we proposed that the interaction between the thiyl radicals produced after NO release and disulfide bonds might contribute to the facilitated matrix degradation of the hybrid MON (Fig. 3D). To verify our hypothesis, we used x-ray photoelectron spectroscopy (XPS) to determine the S_2p_ binding energy of the thiol–disulfide hybrid MON through multi-time-point analysis (0, 12, 24, and 48 h after incubation in SBF) (Fig. 3E to H and Figs. S16 and S17). In the thiol–disulfide hybrid MON, the S_2p_ signal at approximately 162.5 eV was attributed to the thiol group, and the signal at approximately 165.0 eV was attributed to the disulfide bond. After 48 h of incubation, the signal at approximately 168.2 eV from -S-N- disappeared, and the peaks from the disulfide bonds and thiol groups were notably increased, indicating that thiol–disulfide exchange might participate in this process. We further selected a dibenzyl disulfide (named RSSR) as a model molecule to verify the thiol–disulfide exchange by electrospray ionization mass spectrometry (ESI-MS) analysis (Fig. 3I to L and Figs. S18 to S20). After incubating the RSSR with GSNO, we observed the generation of a thiol–disulfide exchange product (chemical formula: C_17_H_20_N_3_O_6_S_2_; molecular weight [MW] = 429.2 Da) and glutathione (GSH), suggesting a rapid thiol–disulfide exchange of disulfide bonds with thiyl radicals (Fig. 3J and Fig. S19). However, there was no thiol–disulfide exchange product after a short incubation of RSSR with GSH (Fig. 3K and Fig. S20). Notably, thiol–disulfide exchange between RSSR and GSH was greatly accelerated with the aid of 2,2-dimethoxy-2-phenylacetophenone [50], a classic radical initiator that triggers the transformation of thiol groups to thiyl radicals (Fig. 3L and Fig. S20). Thus, we believe that thiol–disulfide exchange plays a key role in the dynamic interaction between NO release and the disulfide cleavage-mediated matrix degradation of framework-doped MON-SNO. Taken together, these findings demonstrated that the release of NO produced thiyl radicals, which accelerated matrix degradation through the thiol–disulfide exchange, coordinating the release of Dex and NO from the hybrid silica framework.

### MON-SNO@Dex ameliorates acute colitis in mice

**Fig. 4.**
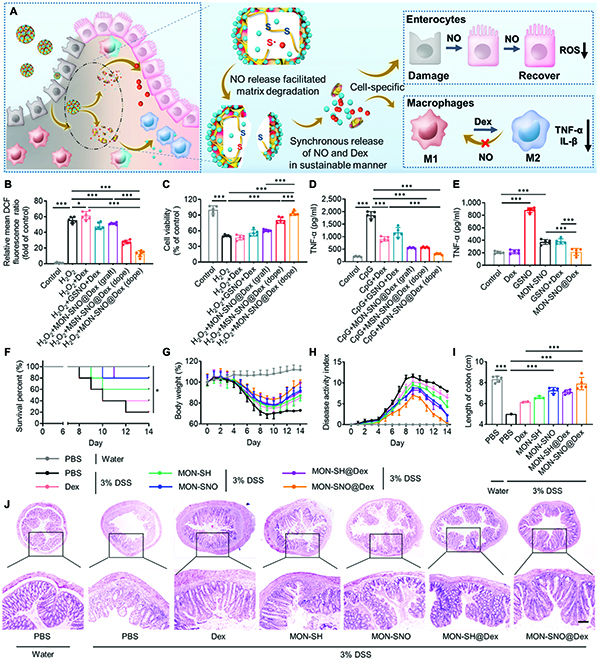
MON-SNO@Dex performed therapeutic effects against DSS-induced acute colitis. (A) Schematic of how MON-SNO@Dex exerted cell-specific therapeutic effects against acute colitis. (B and C) Relative fluorescence intensity of oxidized DCF in HIEC-6 cells (B) and the viability of HIEC-6 cells (C) after incubation with different formulations in the presence of H_2_O_2_ (*n* = 5). (D) RAW 264.7 macrophages were stimulated with different formulations in the presence of CpG (1 μg ml^−1^) (*n* = 5). (E) RAW 264.7 macrophages were stimulated with MON-SNO, GSNO, Dex, GSNO+Dex, and MON-SNO@Dex. Supernatants were assayed for TNF-α by ELISA (*n* = 5). (F to J) C57BL/6 mice were provided with 3% DSS for 7 days. Rectal enema of different formulations on day 3. The survival rate (*n* = 5; *P < 0.05 by Kaplan–Meier survival analysis) (F), daily body weight (G), and DAI changes (H) of mice in each group for 14 days. (I) On day 14, colons in each group were collected and their length was measured (*n* = 5). (J) Representative images of colon sections were stained with H&E. Scale bars: 100 μm. Data were presented as mean ± SD and the statistical significance was calculated via one-way ANOVA with Tukey’s multiple comparisons test. *P < 0.05, ***P < 0.001.

The process of synchronously releasing NO and Dex from framework-doped MON-SNO@Dex and their effect on enterocytes and macrophages in the inflamed colon are presented in Fig. 4A. We investigated the cellular uptake of framework-doped MON-SNO@Dex and subsequent cellular viability. A high degree of intracellular colocalization of MON and the template drug was observed in the endosomes/lysosomes of the cells (Fig. S21). As expected, Dex loading into MON-SNO did not affect the viability of either enterocytes or macrophages (Fig. S22). All Dex-loaded MONs decreased H_2_O_2_-mediated ROS production and toxicity to HIEC-6 cells, while the framework-doped MON-SNO@Dex exhibited the best antioxidative and protective effects (Fig. 4B and C and Fig. S23). As expected, free Dex, free GSNO+Dex, and Dex-loaded MON markedly attenuated CpG oligodeoxynucleotide-triggered TNF-α production in RAW 264.7 macrophages (Fig. 4D and Fig. S24). Consistently, framework-doped MON-SNO@Dex showed markedly reduced macrophage activation compared with surface-grafted MON-SNO@Dex, framework-doped MSN-SNO@Dex, and GSNO+Dex. Importantly, the framework-doped MON-SNO@Dex did not induce the NO-mediated side effect of M1 macrophage polarization (Fig. 4E and Fig. S25).

Having demonstrated that the framework-doped MON-SNO@Dex not only alleviated oxidative damage to enterocytes but also suppressed NO-mediated macrophage activation in vitro, we evaluated the protective effects of MON-SNO@Dex in DSS-induced acute colitis of C57BL/6 mice. In the prevention experiment, mice were fed 3% DSS (w/v) for 7 consecutive days to induce colitis, and then the mice were administered a rectal enema of phosphate-buffered saline (PBS), Dex, MON-SH, MON-SNO, MON-SH@Dex, or MON-SNO@Dex every other day for a total of 5 doses (Fig. S26). Compared to normal mice, DSS-challenged mice exhibited marked body weight loss, an elevated DAI, reduced colon length, and obvious colonic tissue damage (Fig. S26) [41]. In contrast, administration of MON-SNO and MON-SNO@Dex markedly protected the mice and reversed the DSS-induced body weight loss and shortening of the colon on day 10. According to hematoxylin and eosin (H&E) staining, MON-SNO and MON-SNO@Dex also helped maintain the integrity of the colon epithelium and reduced goblet cell depletion and granulocyte infiltration (Fig. S26). Notably, free Dex and MON-SH exhibited markedly weaker protective effects than MON-SNO@Dex. In the therapeutic experiment, a rectal enema was administered every other day starting on day 3 (Fig. S27). Consistent with the preventive effects, MON-SNO@Dex outperformed the other treatments in terms of reducing body weight loss, DAI, and colonic damage, as well as increasing colon length (Fig. 4F to J and Fig. S27). Importantly, 4 of 5 mice died after DSS challenge, while MON-SNO@Dex markedly reduced mortality with a 100% survival rate in this group compared to the survival rates in the MON-SNO (60% survival rate) and Dex (40% survival rate) groups (Fig. 4F). Collectively, MON-SNO@Dex showed the best preventive and therapeutic effects in these acute colitis models.

### MON-SNO@Dex modulates NO in a cell-specific manner in the intestinal microenvironment

**Fig. 5.**
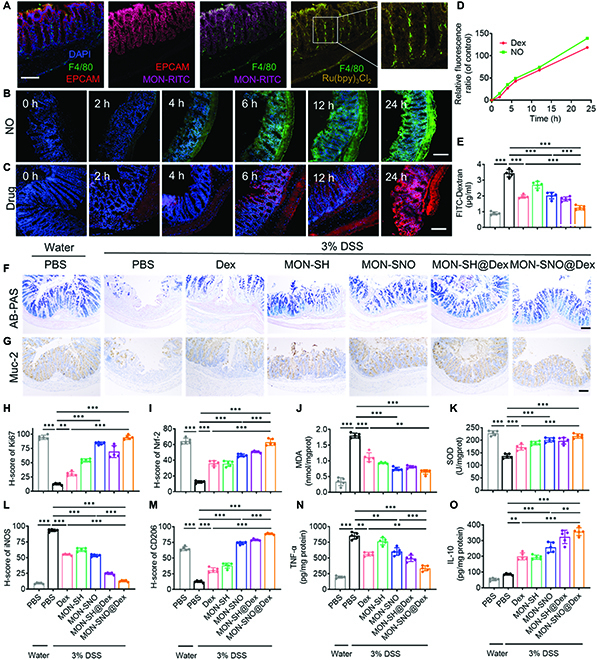
MON-SNO@Dex modulates NO in a cell-specific manner in the intestinal microenvironment. (A) Fluorescent images of colon tissues, which were obtained from mice given 3% DSS water for 3 days and rectal enema administered with MON-RITC@Ru(bpy)_3_Cl_2_ (5 mg kg^−1^) (blue: DAPI, red: EPCAM, green: macrophages, yellow: Ru(bpy)_3_Cl_2_, and pink: MON). Scale bar: 100 μm. (B) Fluorescent images of accumulated NO in DSS-treated inflamed colon. Scale bar: 200 μm. (C) Fluorescent images of accumulated templated drug in DSS-treated inflamed colon. Scale bar: 100 μm. (D) Relative fluorescence intensity of accumulated NO and templated drug in DSS-treated inflamed colon. (E) The content of FITC-Dextran leaked from the intestinal tract in different groups (*n* = 5). (F and G) Representative images of AB-PAS staining and (F) immunohistochemical staining of Muc2 (G) in colonic sections of each group. Scale bars: 100 μm. (H and I) Quantification of Ki67 (H) and Nrf-2 (I) by immunohistochemical staining. (J and K) The colonic protein expression of MDA (J) and SOD (K) from each group. (L and M) Quantification of iNOS (L) and CD206 (M) by immunohistochemical staining. (N and O) The colonic levels of cytokines TNF-α (N) and IL-10 (O) by ELISA in each group (*n* = 5). Data were presented as mean ± SD and the statistical significance was calculated via one-way ANOVA with Tukey’s multiple comparisons test. **P < 0.01, ***P < 0.001.

We next sought to decipher the protective role of MON-SNO@Dex in DSS-induced acute colitis. We developed a dual-fluorescent nanoplatform consisting of rhodamine isothiocyanate (RITC)-labeled MON with the yellow dye Ru(bpy)_3_Cl_2_ as a template drug to determine the distribution and colocalization of the carrier and drug in inflamed colonic tissue (Fig. 5A and Fig. S28). Twelve hours after rectal enema, the fluorescence of the MON primarily overlapped with the signal of epithelial cell adhesion molecule (EPCAM), a biomarker of intestinal epithelial cells. Some of the template drugs accumulated in intestinal macrophages, suggesting that Dex could be delivered to these cells. Next, we quantified the diffusion of the NO and codelivered drugs in the inflamed colon tissue over extended periods of time (Fig. 5B to D). Time-dependently enhanced accumulation and retention of NO and drug were observed in the muscular layer of colonic tissue. The mucosal barrier integrity plays a crucial role in resisting injury, and its function is an important indicator for evaluating the severity of colitis [51]. The serum FITC-Dextran level in DSS-challenged mice was markedly reduced after treatment with MON-SNO@Dex, suggesting that the damaged intestinal mucosa was partially repaired by MON-SNO@Dex (Fig. 5E). We also found that the destruction of colonic goblet cells and collagenous fiber deposition were recovered after the treatment of MON-SNO@Dex (Fig. 5F and Fig. S29). Consistently, the Muc2 protein levels were increased after MON-SNO@Dex treatment (Fig. 5G and Fig. S29). Together, these findings demonstrated that MON-SNO@Dex had a protective effect on the production of colonic mucosal barrier. We further investigated whether MON-SNO@Dex might have a preventative effect by alleviating oxidative stress in enterocytes and regulating the activation of macrophages. We found that MON-SNO@Dex upregulated the expression of Ki67 and markedly downregulated the expression of nuclear factor erythroid 2-related factor 2 (Nrf-2) in colonic tissue (Fig. 5H and I and Fig. S30). Correspondingly, the colonic levels of the oxidative stress markers superoxide dismutase (SOD), malondialdehyde (MDA), catalase (CAT), and glutathione peroxidase (GSH-Px) were reserved after MON-SNO@Dex treatment (Fig. 5J and K and Fig. S30), indicating that MON-SNO@Dex protected enterocytes from oxidative stress.

The innate and adaptive immune cells in the inflammatory microenvironment play a pivotal role in colitis [52]. We found that M1 polarization markers including inducible nitric oxide synthase (iNOS) and CD80 were downregulated, while the M2 polarization marker CD206 was upregulated in the intestinal macrophages of DSS-challenged mice after the treatment of MON-SNO@Dex (Fig. 5L and M and Figs. S30 and S31). Since colitis was classically associated with gut accumulation of pro-inflammatory T-helper 17 (Th17) cells and an insufficient presence of regulatory T cells (Tregs) in the colon [53], we observed a low portion of IL-17-positive cells with a high portion of FoxP3-positive Tregs in colonic tissue after treatment with MON-SNO@Dex (Fig. S31). Consistently, the levels of TNF-α, IFN-γ, IL-6, IL-17A, and IL-1β in the colon of DSS mice were markedly reduced, while the levels of IL-4, IL-22, TGF-β, and IL-10 were correspondingly increased after treatment with MON-SNO@Dex (Fig. 5N and O and Figs. S30 and S31). These results collectively demonstrated that MON-SNO@Dex ameliorated intestinal inflammation by regulating the immune cells in colitis.

**Fig. 6.**
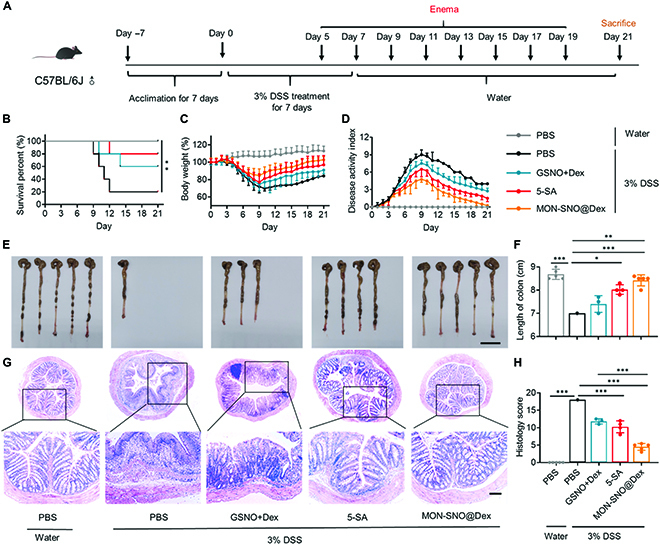
MON-SNO@Dex ameliorated DSS-induced acute colitis in a setting of delayed treatment. (A) C57BL/6 mice were provided with 3% DSS for 7 days. Rectal enema of different formulations on days 5, 7, 9, 11, 13, 15, 17, and 19. (B to D) The survival rate (*n* = 5 mice per group; **P < 0.01 by Kaplan–Meier survival analysis) (B), daily body weight (C), and DAI changes (D) of mice in each group for 21 days (*n* = 5). (E and F) On day 21, colon images of colonic sections in each group (E) and colon length were measured (F). Scale bar: 2 cm. (G and H) On day 21, representative images of colon sections in each group were stained with H&E (G) and colonic damage scores were measured (H). Scale bar: 100 μm. Data were presented as mean ± SD and the statistical significance was calculated via one-way ANOVA with Tukey’s multiple comparisons test. *P < 0.05, **P < 0.01, ***P < 0.001.

Given that many patients experience severe colitis after diagnosis [41], we investigated whether the strategy of NO and Dex codelivery can have positive outcomes when therapy is delayed; therefore, DSS-challenged mice were treated on day 5 and the mice showed high mortality (Fig. 6A). As shown in Fig. 6B to H, 5-SA, a widely used first-line drug in the clinic [4], did not exhibit considerable therapeutic performance. However, MON-SNO@Dex could remarkably protect against DSS-induced death, body weight loss, DAI increase, colon shortening, and colonic damage. The combination of free GSNO and Dex had reduced therapeutic effects compared with MON-SNO@Dex, indicating the advantages of the synchronous release of NO and Dex. To systematically evaluate the safety profile of MON-SNO@Dex, healthy mice were challenged with the nanomaterials according to the current therapeutic dosage (Fig. S32). Mice in all the treatment groups did not display marked changes in body weight and the levels of serum biochemical indicators, including alanine aminotransferase (ALT), aspartate aminotransferase (AST), blood urea nitrogen (BUN), and serum creatinine (CRE). These results were consistent with the negligible pathological changes observed in the major organs (heart, liver, spleen, lung, kidney, small intestine, and colon). In particular, even the high dose of MON-SNO@Dex (10 mg kg^−1^) did not cause any obvious systemic toxicity (Fig. S33).

## Discussion

Although NO-based therapies have shown promise in UC treatment [14,16], the on-demand delivery of NO is challenging, especially when codelivering this gas and small molecules. MON with classic organic–inorganic hybrid architectures have emerged as an intelligent platform to facilitate the controlled delivery of therapeutic agents by integrating the functional versatility of organic components with the stability of the mesoporous silica framework [54,55]. Sophisticated engineering of the hybrid framework can create MON with efficient responsiveness to pH [56], redox [54], light [57,58], temperature [59], and x-rays [60] via matrix degradation, leading to controlled drug release for the management of multiple inflammatory diseases [61,62]. To endow MON with high NO loading and synchronous release properties, we introduced both thiol and disulfide moieties into the framework of silica to fabricate thiol–disulfide hybrid MON. The content and distribution of the thiol groups and disulfide bonds affected NO loading, matrix degradation, and drug release, which are primarily based on 2 factors, the composition (e.g., ratio and type of silane precursors) and synthetic conditions (e.g., temperature and separation mode of precursors), during the hybrid formation process. After systematic investigation, we found that the thiol content of MON-SH increased when increasing the amount of thiol precursors, decreasing the amount of disulfide precursors, or elevating the temperature. MPTES/BTES in a weight ratio of 2:1 produced the thiol–disulfide hybrid MON with the highest thiol content and NO loading. Although the inclusion of BTES somewhat decreased the NO loading, accelerated degradation in SBF is necessary for controlled drug release. Moreover, NO loading markedly increased after the introduction of the organosilica precursor MPTES was appropriately delayed, which might be explained by the specific localization of the active thiol group in the relative outer space of the framework. Having optimized the effects of the composition and synthetic conditions on the thiol group content, we obtained thiol–disulfide hybrid MON integrating high NO loading with matrix-degrading properties.

It is increasingly being recognized that thiol groups are crucial receptors for NO [63]. However, the SNO group-based prodrug GSNO is not stable and quickly releases NO in the body [22,64]. Thus, we incorporated thiol groups in the matrix of MON to facilitate the controlled release of NO. Compared to surface-grafted MON, the framework-doped MON exhibited dramatically higher thiol contents and NO storage while markedly prolonging the burst NO release. We speculated that the unique hybrid architecture of the framework-doped MON that acted as a cage was beneficial to closely tether labile SNO and effectively protect SNO from environmental exposure, further improving the stability of SNO for sustained release. Although thiol-functionalized MSN has been widely used for NO delivery, the presence of thiol groups on their exterior surface has restricted the loading content and stability of NO. In addition to MSN, we included other NO-storing nanomaterials in Table S3, which exhibited lower NO storage (<1 μmol mg^−1^) and faster NO release (<12 h). To the best of our knowledge, our framework-doped MON with thiol–disulfide hybrid frameworks stored the most NO (2.26 μmol mg^−1^) and released NO the slowest (60 h), showing a 10-fold improvement compared with the average parameters among other NO-storing nanomaterials noted. Since intestinal epithelial cells experiencing oxidative damage amplify inflammation in UC, we selected HIEC-6 cells as the target intestinal epithelial cell line to demonstrate the protective effect of MON-SNO against oxidative stress. In addition to the antioxidative activity of MON-SNO (dope), we found that an appropriate dosage of MON-SNO (dope) reduced the pro-inflammatory effect of MON-SNO (graft) on macrophages. As a consequence, the framework-doped MON outperformed the protective effect of both the surface-grafted MON and GSNO due to the higher NO loading and slower NO release, which might rebuild the damaged intestinal barrier and ameliorate inflammation. Given the paradoxical and opposing roles of NO in colitis, the safety and reliability of NO-related treatment have historically remained seemingly difficult and controversial [14,20,21]. In this context, the difference between effective and toxic doses of NO has yet to be determined, which inevitably overshadows its clinical translation. In agreement with previous reports [17,24], we found that low and high doses of GSNO were not more protective than the middle dose. In contrast, MON-SNO with the same amount of NO stored outperformed GSNO and broadened the therapeutic window of NO therapy against colitis. We assume that the underlying reason for this result is that the sustained release of NO in the inflamed colon reduced epithelial poisoning and the risk of macrophage activation. Other nanotherapeutic strategies can widen the therapeutic window mainly by targeting or responding to the characteristics of inflammatory microenvironments [65–70]. Nevertheless, these substantial efforts are still immature and present uncertainties concerning the complexity of inflammation [71,72]. In particular, the sustained release of NO produced via our strategy can be easily achieved regardless of endogenous stimuli, allowing a constant dosage of NO for efficient and safe colitis management.

Given the dual effects of NO as an immunoregulator in inflammation [14,73], accurate regulation of the functions of NO in the inflammatory environment calls for an advanced strategy with both controlled NO delivery and side effect reduction. In such a scenario, NO-mediated enterocyte protection and NO-induced macrophage activation should be balanced simultaneously, which suggests that NO and Dex should be synchronously released in the inflamed intestine. Almost all current nanocarriers exhibit fast NO release and slow drug release, which limits the efficiency and safety of synergistic colitis therapy. We found that the presence of the SNO group facilitated the release of Dex, which led us to ask how NO release affects the degradation of the matrix. Given that thiol–disulfide exchange is one of the most popular dynamic covalent reactions in biological systems [74–77], we hypothesized that thiol–disulfide exchange in the hybrid organosilica framework might facilitate Dex release to coordinate NO release. We observed thiol–disulfide exchange during the NO release process, which led to cleavage of the disulfide bond to accelerate matrix degradation and Dex release. We next indicated that only the thiol group and disulfide bond participated in the thiol–disulfide exchange by comparing the degradation of framework-doped and surface-grafted MON-SNO. Mechanistically, the thiyl radical produced after NO release could interact with the disulfide bond, accelerating the exchange rate. On the basis of these important findings, thiol–disulfide exchanges could be further explored for applications involving the synchronous release of NO and other drugs, not only for inflammation control but also for combined cancer therapy. Such a thiol–disulfide exchange process has not yet been reported with hybrid materials and might open a new door for the development of the next generation of thiol–disulfide hybrid nanoplatforms in biomedicine. A deep understanding of the principle of thiol–disulfide exchange might shed light on how to tailor sophisticated carriers for the synergistic codelivery of 2 compounds based on the thiol group-mediated release of Drug A and the disulfide bond-mediated release of Drug B in the hybrid framework.

The MON-SNO-based synchronous release of NO and Dex not only alleviated oxidative damage in intestinal epithelial cells but also suppressed NO-mediated macrophage activation. We next investigated whether the protective and anti-inflammatory effects of MON-SNO@Dex could be combined in DSS-challenged mice. Here, we demonstrated that MON-SNO@Dex produced better preventative and therapeutic outcomes against acute colitis than both MON-SNO and Dex. We found that after rectal delivery, MON-SNO@Dex reached the inflamed colon and accumulated in both intestinal epithelial cells and macrophages. Subsequently, the sustained release of NO not only reduced oxidative stress but also activated pro-inflammatory macrophages. Moreover, the released Dex exerted potent immunosuppression by polarizing the activated macrophages into the M2 type, indicating that MON-SNO@Dex modulated the functions of NO in a cell-specific manner via the synchronous release of NO and Dex in the intestinal microenvironment. Nonetheless, safety in nanomedicine is paramount when considering clinical applications. In line with our previous study [41], therapeutic doses of MON-SH, MON-SNO, and MON-SNO@Dex did not induce noticeable toxicity, indicating their good safety profiles. On the basis of these findings, our MON-SNO@Dex represents a convenient yet powerful platform by synergistically regulates the inflammatory microenvironment and overcoming the limitations of conventional NO-based therapies. Considering the complexity of NO involvement in colonic inflammation, additional experiments are needed to investigate the effects of other immune cells after MON-SNO@Dex administration before clinical testing.

UC is prevalent worldwide and a well-known cause of colorectal cancer that lacks effective therapy [78]. Despite their serious side effects, lifelong treatment with immunoregulatory drugs is typically the only option for UC management [79–81]. Encouraged by the great therapeutic effects of MON-SNO@Dex in both the preventive and therapeutic models, we finally examined their efficacy in delayed therapy. We orally administered 5-SA, a widely used first-line drug in the clinic, as a positive control for treating severe UC [82]. We found that MON-SNO@Dex exhibited better therapeutic performance than the combination of GSNO and Dex, whereas 5-SA failed to protect animals against severe colitis. Taken together, these results demonstrated that MON-SNO and Dex played critical and complementary roles in the multifaceted benefits of the NO-based combination strategy against colitis, indicating the potential clinical value of MON-SNO@Dex for UC management.

In summary, we markedly improved the storage and sustained release of NO by developing thiol–disulfide hybrid MON, and the resulting MON-SNO broadened the therapeutic window of NO therapy against colitis. Importantly, we demonstrated synchronous release of NO and Dex that occurred by a self-motivated process by which the thiyl radicals produced after NO release cleaved the disulfide bonds to degrade the matrix via thiol–disulfide exchange. Our findings illustrate that the combination of NO and an immunoregulator can profoundly modulate the intestinal microenvironment while exerting potent anti-injury and anti-inflammatory responses to protect and treat DSS-challenged mice. The data in the present work suggest a rational design for thiol–disulfide hybrid MON with on-demand NO loading and release that can be achieved through the coordination of matrix degradation and cargo delivery, which extends the versatility of thiol–disulfide hybrid materials as promising platforms for the synchronous codelivery of bioactive agents, especially in inflammatory diseases and cancer.

## Materials and Methods

### Synthesis of framework-doped MON-SNO

In this work, MON-SH was synthesized by the Stöber process. Cetyltrimethylammonium tosylate (CTAT) was employed as a template, TEOS and BTES were utilized as the inorganic and organic silica precursors. A mixture of CTAT (0.6 g), triethanolamine (TEAH_3_) (0.15 g), and deionized water (40 ml) was stirred at varying temperatures (50, 80, or 95 °C) for 30 min under nitrogen protection. Subsequently, TEOS (1.0, 0.7, or 0.6 g), BTES (0, 0.1, 0.2, or 0.3 g), and ethanol (1.5 ml) were added dropwise into the above system. After 0, 30, 45, or 60 min, a mixed silica precursor of TEOS (0, 0.3, or 0.4 g) and MPTES (0.1, 0.15, 0.2, or 0.3 g) was added dropwise and further reacted for another 4 h. The synthesis of other formulations was achieved by replacing TEOS with tetramethoxysilane (TMOS) or tetrapropoxysilane (TPOS), respectively. The products were collected through centrifugation at 11,000 rpm for a duration of 5 min, and subsequently washed with both water and ethanol. To remove the organic templates, MON-SH was refluxed in an ethanol solution of NH_4_NO_3_ (1% w/v).

The MON-SNO was synthesized according to published procedures with slight changes [83]. MON-SH (20 mg) and an excess t-butyl nitrite were added to the mixed solution of toluene/methanol (v/v = 9:1, 20 ml) and allowed to react for 10 h. MON-SNO was collected through centrifugation and washed with chilled methanol. MON-SNO was stored at −20 °C in vacuo until further use.

### Measurement of active thiol group contents using Ellman’s reagent

The content of the active thiol group was assessed using a chemical test based on the DTNB reagent (5,5- dithiobis-[2-nitrobenzoic acid]) [84]. At first, sodium acetate (50 mM) and DTNB (2 mM) were dissolved in water (solution A). Tris (1 M) was dissolved in water and adjusted to pH 8.0 (solution B). Subsequently, solution A (50 μl), solution B (100 μl), and water (840 μl) were carefully mixed. Finally, 10 μl of MON-SH (1 mg ml^−1^) was added into the above-mixed solution (990 μl) and further reacted for 20 min, and analyzed using a UV–Vis spectrophotometer. A standard calibration curve was generated using L-cysteine.

### In vitro drug release

A NO-sensitive fluorophore, Griess reagent was used to measure the release kinetics of NO [85]. GSNO, MON-SNO (graft), and MON-SNO (dope) (0.02 mM with equal NO concentration) were dispersed in PBS solution (pH 7.4) and incubated at 37 °C (100 rpm). At predetermined time intervals, 50 μl of the supernatant was withdrawn and reacted with Griess Reagent I (50 μl) and Griess Reagent II (50 μl) for 5 min. The concentration of nitrite was calculated using a standard curve of sodium nitrite with a SpectraMax M5 microplate reader. To examine if the cage architecture is universal and expandable, the NO release kinetics of MON-SNO (graft) and MON-SNO (dope) was studied under different temperatures (4, 10, 25, and 37 °C), pH values (7.4 and 8), and shaking speeds (50, 100, and 300 rpm) by the same processes in every context.

To evaluate the release of both Dex and NO, MON-SNO@Dex (dope), MON-SNO@Dex (graft), MON-SH@Dex (dope), and MSN-SNO@Dex (dope) were dispersed in PBS (pH 7.4) and incubated at 37 °C (100 rpm). At predetermined time intervals, the released amounts of Dex and NO were measured by UV–Vis spectroscopy and a SpectraMax M5 microplate reader.

### Thiol–disulfide exchange mediated degradation

MON-SNO@Dex (dope), MON-SNO@Dex (graft), MON-SH@Dex (dope), or MSN-SNO@Dex (dope) (100 μg ml^−1^) was incubated in SBF (100 rpm). At different time intervals, each sample was collected, the morphology was evaluated by a transmission electron microscope (TEM), and the released Si content in the supernatant was detected by ICP-MS. Additionally, XPS was used to determine the S_2p_ binding energy.

### Verify the thiol–disulfide exchange at the molecular level

Dibenzyl disulfide (10 mM) was mixed with GSNO (2 mM) in 10 ml of methanol/H_2_O (v/v = 9:1) and reacted for 0.5, 3, and 6 h. As compared, dibenzyl disulfide (10 mM) was mixed with GSH (2 mM) in 10 ml of methanol/H_2_O (v/v = 9:1) and reacted for 6 h. In addition, dibenzyl disulfide (10 mM) and GSH (2 mM) were mixed with 2,2-dimethoxy-2-phenylacetophenone (10 mM or 30 mM) in 10 ml of methanol/H_2_O (v/v = 9:1) and then reacted for 6 h. The structural analysis of the thiol–disulfide exchange reaction products was carried out using ESI-MS.

### Detection of intracellular NO accumulation

The intracellular NO accumulation in both HIEC-6 cells and RAW 264.7 macrophages was investigated using a NO-specific probe, DAF-FM DA [38]. DAF-FM DA, upon entering the cell membrane, is hydrolyzed by intracellular esterase to yield DAF-FM. DAF-FM showed no fluorescence initially; a reaction with NO would produce benzotriazole with high fluorescence. Cells were seeded into dishes at 2 × 10^5^ cells and cultured overnight. MON-SNO (dope), MON-SNO (graft), and GSNO (0.226 mM with equal NO concentration) were added into the dishes and incubated for 2, 4, 6, 12, and 24 h. The cells were washed with PBS and further incubated with DAF-FM DA (10 μΜ) for 30 min. Photomicrographs were acquired using CLSM.

### Pro-inflammatory and anti-inflammatory assay in vitro

RAW 264.7 macrophages were seeded in 96-well plates (1 × 10^4^ cells/well) and cultured overnight. The cells were treated with GSNO, MON-SNO (graft), MON-SNO (dope), Dex, GSNO+Dex, and MON-SNO@Dex (dope) at the indicated concentrations (0.226 mM with equal NO concentration) for 24 h, with the untreated cells as control groups. In addition, the cells were treated with CpG (0 or 1 μg ml^−1^) with medium containing MON-SNO@Dex (graft), MSN-SNO@Dex (dope), Dex, GSNO+Dex, MON-SH, MON-SNO, Dex, MON-SH@Dex, and MON-SNO@Dex (dope) for 24 h, respectively. Subsequently, the supernatant was collected and centrifuged (10,000 *g*, 10 min) at 4 °C to pellet down any cell. TNF-α, IL-1β, IL-6, IL-4, or IL-10 was determined by enzyme-linked immunosorbent assay (ELISA) following the manufacturer’s instruction.

### In vitro ROS assay

To examine the intracellular ROS level, the probe H_2_DCFDA was utilized [38]. Firstly, HIEC-6 cells were seeded in 6-well plates and incubated overnight. The cells were pre-incubated with 100 μM H_2_O_2_ for 4 h and the culture medium was replaced with GSNO, MON-SNO (graft), MON-SNO (dope), MSN-SNO, Dex, GSNO+Dex, MON-SNO@Dex (graft), MSN-SNO@Dex (dope), and MON-SNO@Dex (dope) at the indicated concentrations (0.226 mM with equal NO concentration). Subsequently, the cells were rinsed with PBS and incubated with H_2_DCFDA (10 μM) for 20 min. Photomicrographs were acquired using CLSM.

### Animals and treatment

Male C57BL/6 mice (6 weeks old) were obtained from Hunan SJA Laboratory Animal Co., Ltd. All animals received care in compliance with the guidelines outlined in the *Guide for the Care and Use of Laboratory Animals* and the procedures (ACE2019031) were approved by the South China University of Technology Animal Care and Use Committee.

For the therapeutic window experiment, the C57BL/6J mice were randomly divided into 7 groups (5 per group). Mice were administered with 3% (w/v) DSS (MW = 36,000 to 50,000) water solution for 7 days to induce acute UC. On days 0, 2, 4, 6, and 8, the mice were fasted for 8 h and were administered a rectal enema of PBS, GSNO, and MON-SNO at the same dosage of NO (100 μl of hydrogel, 1.13, 11.3, and 33.9 μmol kg^−1^), respectively. During this period, mice were monitored daily to determine the DAI. On day 10, mice were sacrificed and the colon was excised.

The prevention experiment was performed under the same experimental conditions as the therapeutic window experiment. On days 0, 2, 4, 6, and 8, the mice were fasted for 8 h and were administered a rectal enema of PBS, MON-SH, MON-SNO, Dex, MON-SH@Dex, and MON-SNO@Dex (100 μl of hydrogel, equivalent Dex dose of 1 mg kg^−1^, and equivalent MON dose of 4 mg kg^−1^), respectively. The mice were sacrificed on day 10. Additionally, the mice were fasted for 8 h and then received oral administration of FITC-Dextran at 600 mg/kg. After 3 h of treatment, blood was collected and centrifuged at 2,000 *g* for 20 min, and the fluorescence intensity of supernatants was measured. This study investigated several markers associated with colonic oxidative stress in UC, including SOD, MDA, CAT, and GSH-Px. The colon sections were stained with H&E and alcian blue and periodic acid–Schiff (AB-PAS). Immunohistochemistry was performed to assess expression levels of Ki67, Nrf-2, iNOS, CD206, F4/80, and Muc2. To examine colonic immune cells when treated by MON-SNO@Dex with or without colitis, the healthy mice were fasted for 8 h and MON-SNO@Dex was administered via enema at the same dosage. Immunofluorescence was performed to assess expression levels of CD80. Immunohistochemistry was performed to assess expression levels of IL-17 and FoxP3. Furthermore, ELISA tests were also conducted to measure levels of IL-6, IL-4, IL-10, IL-1β, TNF-α, IFN-γ, IL-17A, TGF-β, and IL-22. More details of the characterization and description can be found in the Supplementary Materials.

For the therapeutic experiment, treatment was administered starting on day 3. The mice were fasted for 8 h and received rectal enemas of PBS, MON-SH, MON-SNO, Dex, MON-SH@Dex, and MON-SNO@Dex (100 μl of hydrogel, equivalent Dex dose of 1 mg kg^−1^, and equivalent MON dose of 4 mg kg^−1^) every other day. The mice were sacrificed on day 14.

For the delayed therapy, treatment was administered starting on day 5. The mice were fasted for 8 h and received rectal enemas of PBS, free GSNO, Dex, and MON-SNO@Dex, compared with oral administration of 5-SA at 5 mg kg^−1^ every other day. The mice were sacrificed on day 21.

### Immunofluorescence staining

Mice were administered 3% DSS in drinking water for 3 days. The mice were fasted for 8 h and received a single enema delivery of MON-RITC@Ru(bpy)_3_Cl_2_ (5 mg kg^−1^). At 12 h post-administration, the colon tissue was excised and embedded in optimal cutting temperature compound. Colon tissue sections were prepared with a thickness of 5 μm. These sections were air-dried for at least 1 h and then fixed in acetone at −20 °C for 20 min. After blocking with 8% bovine serum albumin, the sections were incubated with FITC-conjugated anti-F4/80 (dilution 1:1,000) and Alexa Fluor 647-conjugated anti- EPCAM (dilution 1:1,000). Finally, the sections of colon tissue were stained with DAPI before observation.

### Statistical analysis

Statistical analyses were performed using Prism 7 software (GraphPad Software, La Jolla, CA), and values were expressed as means ± SD. Statistical analysis of variance (ANOVA) followed by Tukey’s multiple comparisons was used to compare different treatment groups. Comparison of Kaplan–Meier survival curves was performed with the log-rank Mantel–Cox test. Differences were regarded as statistically significant with *P < 0.05, **P < 0.01, and ***P < 0.001.

## Data Availability

All data needed to evaluate the conclusions in the paper are present in the paper and/or the Supplementary information.
