# Supplementary material for: Thiol–Disulfide Exchange Coordinates the Release of Nitric Oxide and Dexamethasone for Synergistic Regulation of Intestinal Microenvironment in Colitis
Source: Research (Wash D C). 2023 Aug 1;6:0204. doi: 10.34133/research.0204 (PMC10393581; doi:10.34133/research.0204)
Supplement: Supplementary 1 — Supplementary Materials and Methods Figs. S1 to S33 Tables S1 to S3 [file research.0204.f1.docx]

Supplementary Materials for

**Thiol-disulfide Exchange Coordinates the Release of Nitric Oxide and Dexamethasone for Synergistic Regulation of Intestinal Microenvironment in Colitis**

Junna Lu^1,2^, Tongfei Shi^1,2^, Chengxin Shi^4^, Fangman Chen^1*^, Chao Yang^1,3^, Xiaochun Xie^5^, Zheng Wang^6^, He Shen^6^, Jiaqi Xu^7^, Kam W. Leong^3*^ & Dan Shao^1,2,8,9*^

^1^ School of Biomedical Sciences and Engineering, South China University of Technology, Guangzhou International Campus, Guangzhou, Guangdong 510006, China

^2^ National Engineering Research Center for Tissue Restoration and Reconstruction, South China University of Technology, Guangdong 510006, China

^3^ Department of Biomedical Engineering, Columbia University, New York, NY 10027, USA

^4^ Department of Plastic and Aesthetic Center, The First Affiliated Hospital of Zhejiang University, Hangzhou 310000, China

^5^ School of Medicine, South China University of Technology, Guangzhou, Guangdong 510006, China

^6^ CAS Key Laboratory of Nano-Bio Interface, Suzhou Institute of Nano-Tech and NanoBionics, Chinese Academy of Sciences, Suzhou 215123, China

^7^ CAS Key Laboratory for Biomedical Effects of Nanomaterials & Nanosafety, CAS Center for Excellence in Nanoscience, National Center for Nanoscience and Technology, Beijing 100190, China

^8^ Guangdong Provincial Key Laboratory of Biomedical Engineering, South China University of Technology, Guangzhou 510006, China

^9^ Key Laboratory of Biomedical Materials and Engineering of the Ministry of Education, South China University of Technology, Guangzhou 510006, China

*Correspondence authors: [stanauagate@outlook.com](mailto:stanauagate@outlook.com) (D. Shao); chenfangman@hotmail.com (F. Chen); [kam.leong@columbia.edu](mailto:kam.leong@columbia.edu) (K. W. Leong)

**Supplementary Methods**

**Materials and Reagents**

Tetramethoxysilane (TMOS), tetraethylorthosilicate (TEOS), tetrapropoxysilane (TPOS), bis(triethoxysilylpropyl) disulfide (BTES), 3-mercaptopropyltriethoxysilane (MPTES), cetyltrimethylamium tosylate (CTAT), triethanolamine (TEAH_3_), 3-aminopropyltriethoxysilane (APTES) were purchased from Adamas-beta (Shanghai, China). Rhodamine B Isothiocyanate (RITC), sulforhodamine B (SRB), ammonium nitrate (NH_4_NO_3_), sodium nitrite (NaNO_2_), hydrogen peroxide (30% H_2_O_2_), dibenzyl disulfide, 2,2-Dimethoxy-2-phenylacetophenone, S-nitrosoglutathione (GSNO), glutathione (GSH), dexamethasone (Dex), doxorubicin (DOX), and anhydrous ethanol were purchased from Beijing Chemical Reagent Co. (Beijing China). 5,50-Dithiobis-(2-nitrobenzoic acid) (DTNB, Ellman’s reagent), Ru(bpy)_3_Cl_2_, hydrochloric acid (HCl, 37%), L-cysteine, Poloxamer 407 (MW 14,600), tris(hydroxymethyl)aminomethane (Tris, ≥99.5%), and fluorescein isothiocyanate conjugated dextran (FITC-Dextran) were purchased from Sigma-Aldrich (St. Louis, MO, USA). 3-Amino,4-aminomethyl-2′,7′-difluorescein, diacetate (DAF-FM DA), phosphate buffer solution (PBS), LysoTracker Green DND-99, 4',6-diamidino-2-phenylindole (DAPI), 2′,7′-dichlorofluorescin diacetate (DCFH-DA), Griess reagent were purchased from Beyotime Biotechnology (Shanghai, China). Simulated body fluid (SBF) was purchased from Leagene Biotechnology (Beijing, China). Dulbecco's modified Eagle's medium (DMEM), fetal bovine serum (FBS), 0.25% trypsin-EDTA and 1% penicillin-streptomycin were purchased from Gibco Co., Ltd. (Carlsbad, CA, USA). The water with a resistivity of 18.2 MΩ·cm was used in all of the experiments.

Dextran sulfate sodium (DSS) (MW 36,000-50,000) was purchased from MP Biomedicals (Santa Ana, California, US). CpG-oligodeoxynucleotide was purchased from Sangon Biotech Co., Ltd. (Shanghai, China). Tumor necrosis factor-α (TNF-α), interleukin-1β (IL-1β), interleukin-6 (IL-6), interleukin-10 (IL-10), interleukin-4 (IL-4), interleukin-17A (IL-17A), interleukin-22 (IL-22), [interferon-γ (IFN-γ)](http://www.baidu.com/link?url=TajFoZ32OyKhbpbtV-Y8DEawC2dMpyMbaE55is-tqTyKFMr9nchNGgdSJ1oy59AVBTmQlXxOOHyIdaVm44sr7UyXdtoG0bcD1vjA32UOBYL38aP9wAEwBEnWnCauJdCIzfnBZvv4pPB8W0lprYsuItwj_LaFX9f06UtD2izqSyvq6q2R-6aNSJVttWHSHzJD_c_tQs_HmOZrtPRjpdvqIBiPTrdZM4iKNSmKch2S_L2QQmidYwoh5n4Zf82Mo4y52xxTjmNoWDpPcbRsTazPJMix5FexiniGrctvey49qODOLUoVPV_LihK0pS5Jv9c0LcTg9Ke8ycvPaqNrrCdvxWdFnItFe7yjg0_dAIgOvnY-zHyLtHerzQj1SMP4fkSis6osO6W4EbnfEZIf59_Fx1p43aItJzaT0E-SQwV6P9xqlQpjT1XKq8WCckfXD0ASf6CVf49EGrs3-Ox4_Xb2dJFFrwxtTBvR6gOTRxe7Po8dGB6fDheuc4rWz3XMyPSjOIsikKjLlENmwZX344HUMa) and transforming growth factor-β (TGF-β) were purchased from Elabscience Biotechnology Co., Ltd (Wuhan, China). Malondialdehyde (MDA) assay kit, catalase (CAT) assay kit, superoxide dismutase (SOD) assay kit, and glutathione peroxidase (GSH-Px) assay kit were purchased from Nanjing Jiancheng Bioengineering Institute (Nanjing, China). Anti- F4/80 antibody, anti-Ki67 antibody, anti-CD206 antibody, anti-Nrf-2 antibody, anti-iNOS antibody, anti-Muc2 antibody, anti-IL-17 antibody and anti-FoxP3 antibody were purchased from Abcam (Cambridge, UK). Alcian blue and periodic acid Schiff (AB-PAS) was acquired fromServicebio Technology (Wuhan, China). Alexa Fluor 647-conjugated anti-epithelial cell adhesion molecule (EPCAM), FITC-conjugated anti- F4/80 were purchased from BioLegend (San Diego, CA, USA). BCA protein assay kit was purchased from Thermo Fisher Scientific, Inc (Waltham, MA, USA).

**Characterization**

The morphologies of the nanomaterials were characterized with a JEM-2100F transmission electron microscope (TEM, JEOL, Ltd., Japan) and a scanning electron microscope (SEM, FEI Quanta 200F, US). The hydrodynamic diameter and zeta potential of the nanomaterials were characterized with a Nano-ZS 90 Nanosizer (Malvern Instruments Ltd., Worcestershire, UK). Nitrogen isotherm data were collected on a Micromeritics ASAP 2020. Specific surface area and pore size distributions were evaluated and calculated by the Brunauer-Emmett-Teller (BET) and Barrett-Joyner-Halenda (BJH) methods. The FTIR spectra of the nanomaterials were recorded using an FTIR spectrophotometer (Bruker, Germany) in the scan range of 400-4000 cm^-1^. UV–vis absorption spectra were obtained on a UV-2600 spectrometer (Shimadzu Ltd., Japan). The Si and S element content of nanomaterials was measured by inductively coupled plasma mass spectrometry (ICP-MS) (Aurora, M90, US). High-resolution mass spectra were acquired on a Waters Q-TOF Ultima ESI mass spectrometer or a Waters Synapt G2-Si ESI/LC-MS spectrometer. The compositions and chemical species were analyzed by X-ray photoelectron spectroscopy (XPS) (Thermo Scientific K-Alpha spectrometer, US). The experimental peaks were fitted with Avantage software. Fluorescence images were acquired using a confocal laser scanning microscope (CLSM) (LSM 880, Fast Airyscan, Zeiss, Germany). Flow cytometry was analyzed by FongCyte (Beijing Challen Biotechnology Co., Ltd). Cell viability was tested by SpectraMax M5 microplate reader (Molecular Devices, Silicon Valley, CA, US). Biochemical parameters, including alanine aminotransferase (ALT), aspartate aminotransferase (AST), blood urea nitrogen (BUN), and serum creatinine (CRE), were analyzed automatically using Coulter LX2D instrumentation (Beckman, Brea, CA).

**Synthesis of MON-SH (graft)**

To demonstrate the advantages of a framework-doped strategy for the introduction of the thiol group, MON-OH with grafting thiol group was prepared. Firstly, MON-OH was prepared. Subsequently, the MON-OH (100 mg) was dispersed in ethanol and MPTES (200 µL) was added dropwise into the above system. After stirring at 80°C for 12 h, the MON-SH (graft) was washed with ethanol and water.

**Synthesis of dye-labeled MON**

For tracking cellular internalization, MON-RITC was prepared with slight modifications according to the method we have previously reported[^1^](#_ENREF_1). Firstly, RITC-APTES was synthesized by suspending 5 mg of RITC in 4 mL of ethanol and adding 1 mL of APTES dropwise. Subsequently, 100 mg MON-SH was dispersed in 100 mL ethanol under sonication. The solution was stirred at 80 °C for 3 h and cooled to room temperature, RITC-APTES (100 μL) was added dropwise into the above system. The mixture was stirred at room temperature for 1 h and further refluxed at 80 °C for 12 h. The synthesized MON-SH-RITC (denoted as MON-RITC) were collected, washed, and dried for further experiments.

**Synthesis of MON@Dex, MON-RITC@DOX and MON-RITC@Ru(bpy)_3_Cl_2_**

To load Dex, Dex was added to the solution of MON (2 mg/mL) and stirred for 24 h. The resultant mixture was washed and collected as MON@Dex for further use. The supernatant was collected for calculating the drug-loading content by using the equation, drug loading content (%) = mass of Dex in MON@Dex/ mass of MON@Dex.

For loading DOX and Ru(bpy)_3_Cl_2_, DOX or Ru(bpy)_3_Cl_2_ (30 mg) and MON-RITC (60 mg) were dispersed in 10 mL DMSO and stirred for 24 h. The resulting product was washed thrice for further use.

**Cell culture**

Human intestinal epithelial cell line-6 (HIEC-6 cells), and RAW 264.7 murine macrophage cell line (RAW 264.7 macrophages) were obtained from American Type Culture Collection (Manassas, NJ, USA). All the media were supplemented with 90% DMEM, 10% FBS, and 1% penicillin-streptomycin. These cell lines were cultured at 37 °C in a humidified and 5% CO_2_ atmosphere. When adherent cells reached ~ 60 to 70% confluence, they were detached with 0.25% trypsin-EDTA growth medium to allow for continued passaging.

**Endocytosis of nanomaterials**

To investigate the cellular internalization of MON-RITC@DOX, RAW 264.7 macrophages were seeded in a 6-well plate (2×10^5^ cells/well) and cultured overnight. The cells were incubated with MON-RITC@DOX at a concentration of 50 μg mL^-1^. After 3 h of incubation, the cells were stained with LysoTracker Green DND-99 for 30 min and DAPI for 10 min. Subsequently, the cells were washed with PBS and fluorescence images were obtained by CLSM.

**Cell viability**

The cell viability was assessed using the SRB assay, which was conducted in accordance with a previous study[^2^](#_ENREF_2). Briefly, RAW 264.7 macrophages and HIEC-6 cells were seeded in 96-well plates (1×10^4^ cells/well). The next day, cells were treated with different nanomaterials (0-2.26 mM with equal NO concentration of MON-SNO (dope), MON-SNO (graft) and GSNO; equivalent to 0-1000 μg mL^-1^ MON or 0-250 μg mL^-1^ Dex). The cells were further cultured for 6, 12, 24 and 48 h before SRB assay. The optical density (OD) at the wavelength of 570 nm was determined to calculate viability.

To determine the protective effect of NO from oxide damage, HIEC-6 cells were seeded in 96-well and treated with 1000 µM H_2_O_2_ for 4 h. GSNO, MON-SNO (graft), MON-SNO (dope), MSN-SNO, Dex, GSNO+Dex, MON-SNO@Dex (graft), MSN-SNO@Dex(dope) and MON-SNO@Dex(dope) (0.226 mM with equal NO concentration) were added, and the cells were further incubated for 24 h. Additionally, different concentrations of MON-SNO, Dex, and MON-SNO@Dex were also added and the cells were incubated for 24 h.

**Synthesis of hydrogels**

In the treatment of ulcerative colitis (UC), the administration route can significantly affect the therapeutic efficacy. Local administration via rectal enema is a preferred approach, as it not only avoids the potential physiological toxicity of drug carriers entering the bloodstream, but also increases the drug utilization efficiency[^3^](#_ENREF_3). Hydrogels can enhance drug adhesion to the colon, and poloxamer 407 (Pluronic F-127) is a safe polymer approved by the U.S. FDA for such applications. In this study, we synthesized Pluronic F-127 hydrogels with a mass concentration of 28%. F127 hydrogel (28%) is a flowing liquid phase at 4℃, which transforms into a homogeneous low-viscosity liquid phase upon heating to approximately 37℃, the phase transition behavior maximizes drug retention rates. In the DSS-induced colitis model, mice received rectal enemas of drugs with the aid of the F127 hydrogel (28%).

**Analysis of cytokines and** **redox level**

To measure the cytokine levels in the colon tissue, 100 mg of colon tissue was homogenized and centrifuged to obtain the supernatant. TNF-α, IFN-γ, IL-6, IL-4, IL-10, IL-17A, IL-22, TGF-β and IL-1β were measured using the ELISA technique as per the instructions of the manufacturer. The colonic redox levels were determined by MDA, CAT, SOD and GSH-Px assay kit.

**Disease activity index**

The mice were monitored throughout the course of therapy, and disease activity index (DAI) was calculated to assess weight loss, stool consistency and rectal bleeding according to a published method[^4^](#_ENREF_4), where DAI was increased in the colitic mice. DAI was calculated as the sum of the scores of stool consistency (0: hard, 2: soft, 4: diarrhea), fecal occult blood using Hemoccult Sensa (0: negative, 2: positive, 4: macroscopic) and weight loss (0: <1%, 1: 1-5%, 2: 5-10%, 3: 10-20%, 4: >20%). The disease score was calculated as the average of these three parameters.

**Histology**

Colon tissue was fixed with paraformaldehyde (4%), dehydrated, embedded in paraffin, sectioned with 5 μm, and then stained with hematoxylin and eosin (H&E). A digital microscope was used for pathological examination. The tissue damage score was evaluated according to the previous report[^5^](#_ENREF_5). Histological examination was performed by two independent observers on H&E slides of paraffin colon sections. Histology score was assessed for severity of inflammation (0: none, 1: slight, 2: moderate, 3: severe), PMN infiltration/HPF (0: <5, 1: 5-20, 2: 21-60, 3: 61-100, 4: >100), depth of injury (0: none, 1: mucosa, 2: mucosa and submucosa, 3: transmural), crypt damage (0: none, 1: basal 1/3, 2: basal 2/3, 3: only surface epithelium intact, 4: entire crypt lost), and adjusted to tissue involvement by multiplication of percentage factor (x1: 0-25%, x2: 26-50%, x3: 51-75%, x4: 76-100%).

**Alcian blue and periodic acid-schiff (AB-PAS) staining.**

Paraffin-embedded colon specimens of 4 μm thickness were analyzed for goblet cells by AB-PAS staining. Samples were stained with Alcian Blue staining solution, treated with 1% aqueous periodic acid, and stained with Schiff’s reagent, and then, nuclei were lightly stained with hematoxylin. Samples were differentiated with acid alcohol, washed with Scott’s tap water, and then finally dehydrated, cleared, and mounted on glass slides with coverslips.

**Immunohistochemical**

The colon tissue of different groups were deparaffinized and hydrated. Briefly, slides were baked for 1.5 h at 90 °C, and then dewaxed with xylene and rehydrated through a graded series of ethanol solutions (100%, 90%, and 70%) and distilled water. Microwave treatment was conducted with ethylenediaminetetraacetic acid (EDTA) or citrate buffer to retrieve antigen, followed by blocking with 1% (w/v) BSA solution for 15 min at room temperature. The immunohistochemistry was accomplished with the CD206 (dilution 1:200), Nrf-2 (dilution 1:500), iNOS (dilution 1:800), Ki67 (dilution 1:500), F4/80 (dilution 1:500), Muc2 (dilution 1:500), IL-17 (dilution 1:500) and FoxP3 antibody (dilution 1:500).

Evaluation of immunohistochemical (IHC) staining was carried out by two independent pathologists. Results were evaluated the stained slides as no staining according to the previous report[^6^](#_ENREF_6): 0, weak: 1+, moderate: 2+, and strong staining: 3+. The cells remaining positively stained were estimated as percent (0%–100%). An H-score was generated in 0–300 range by multiplying the percentage of positively stained cells and staining intensity score (H-score= 3 × the corresponding positive percentage + 2 × the corresponding positive percentage + 1 × the corresponding positive percentage + 0 × the corresponding positive percentage).

***In vivo* drug release.**

Mice were administered 3% DSS in drinking water for 3 days. The mice were fasted for 8 h and received a single rectal enema of MON-SNO@DOX (5 mg kg^-1^). At 2, 4, 6, 12, and 24 h post-treatment, rectal enema of DAF-FM DA were administered. Mice were subsequently sacrificed and colon tissue was embedded in OCT compound. The sections of colon tissue were stained with DAPI before observation.

**Biosafety experiment**

In the biosafety experiment, healthy mice were utilized to evaluate the safety profiles of different formulations. The healthy mice were randomly divided into seven groups (five per group) and rectal enema of PBS, MON-SH, MON-SNO, Dex, MON-SH@Dex and MON-SNO@Dex (100 µL hydrogel, Dex: 1 mg kg^-1^, MON: 4 mg kg^-1^) every other day for a total of 6 doses. In addition, healthy mice were treated with MON-SNO@Dex at a high dose (100 µL hydrogel, 10 mg kg^-1^). On day 40, all the mice were sacrificed, and H&E staining was performed on the liver, lung, heart, spleen, kidney, [small](javascript:;) [intestine](javascript:;) and colon. Biochemical parameters including AST, ALT, BUN, and CRE were also evaluated by collecting serum samples from the mice via orbital sinus blood centrifugation at 3000 g for 15 min.


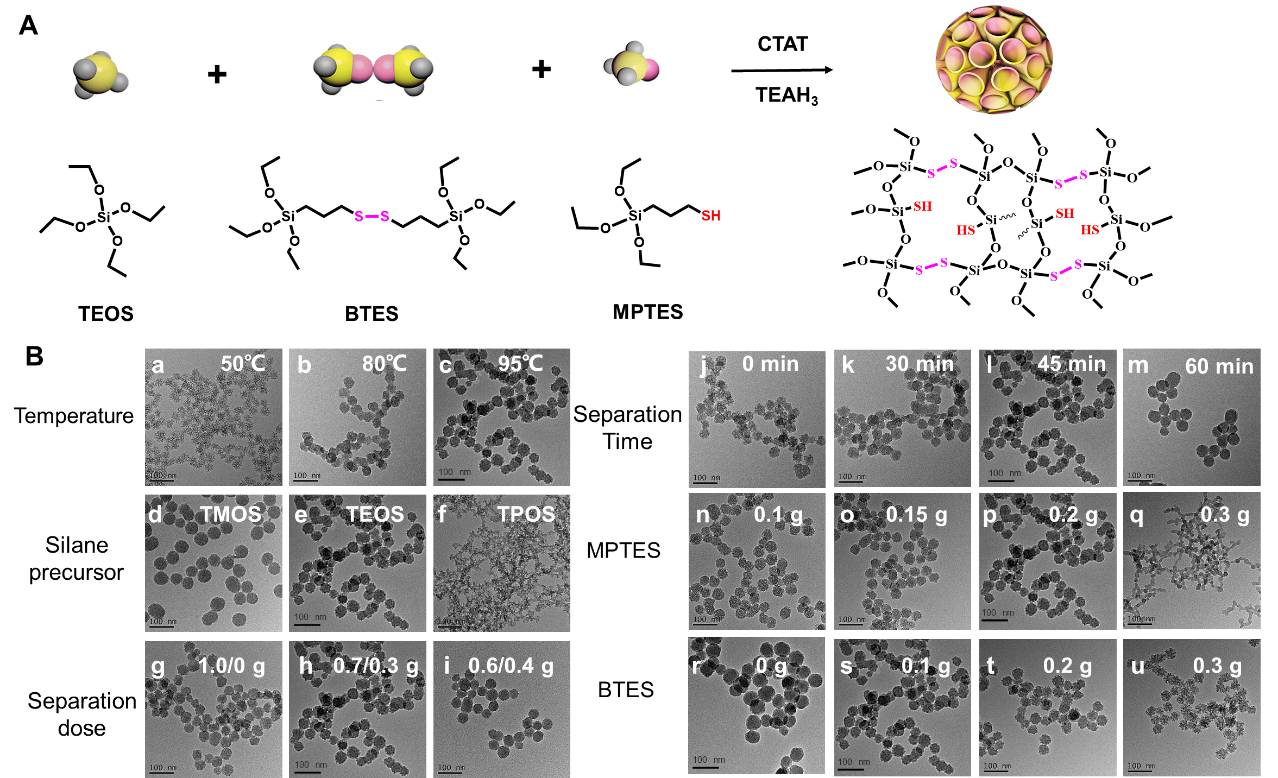


**Supplementary Figure 1.** (**A**) Schematic diagram for preparation of the MON-SH. (**B**) TEM images of MON-SH. MON-SH was synthesized at various temperatures of 50℃, 80℃ and 95℃ (a−c, respectively). MON-SH was synthesized by using various inorganic silica precursors of TMOS, TEOS and TPOS (d−f, respectively). MON-SH was synthesized by using different separation doses and added TEOS twice at varied weight ratios of 1.0/0 g, 0.7/0.3 g and 0.6/0.4 g (g−i, respectively). MON-SH was synthesized by MPTES and TEOS at different separation times of 0 min, 30 min, 45 min, and 60 min (j−m, respectively). MON-SH was synthesized with various MPTES amounts of 0.1 g, 0.15 g, 0.2 g and 0.3 g (n−q, respectively). MON-SH was synthesized with various BTES amounts of 0 g, 0.1 g, 0.2 g and 0.3 g (r−u, respectively). Scale bars: 100 nm.


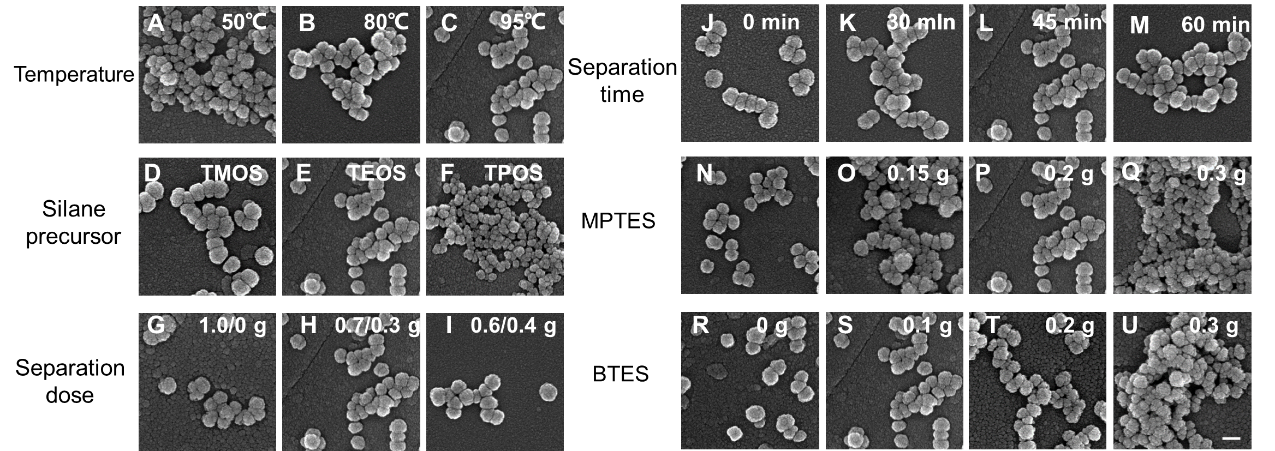


**Supplementary Fig. 2.** SEM images of MON-SH. (**A** to **C**) MON-SH was synthesized at various temperatures of (A) 50℃, (B) 80℃ and (C) 95℃. (**D** to **F**) MON-SH was synthesized by using various inorganic silica precursors of (D) TMOS, (E) TEOS and (F) TPOS. (**G** to **I**) MON-SH was synthesized by using different separation doses and added TEOS twice at varied weight ratios of (G) 1.0/0 g, (H) 0.7/0.3 g and (I) 0.6/0.4 g. (**J** to **M**) MON-SH was synthesized by adding MPTES and TEOS at different separation times of (J) 0 min, (K) 30 min, (L) 45 min, and (M) 60 min. (**N** to **Q**) MON-SH was synthesized with various MPTES amounts of (N) 0.1 g, (O) 0.15 g, (P) 0.2 g and (Q) 0.3 g. (**R** to **U**) MON-SH was synthesized with various BTES amounts of (R) 0 g, (S) 0.1 g, (T) 0.2 g and (U) 0.3 g. Scale bars: 100 nm.


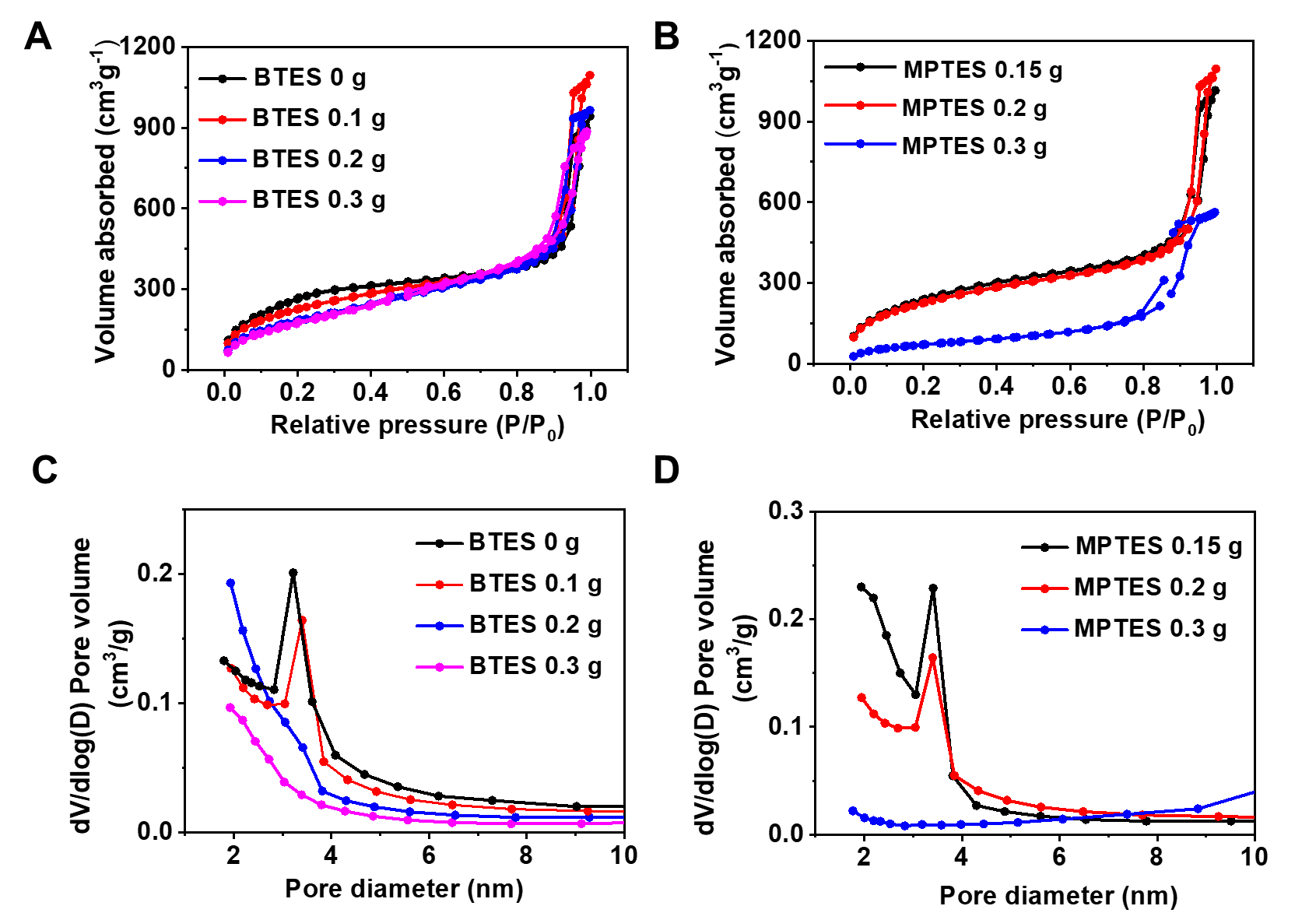


**Supplementary Fig. 3.** (**A**, **B**) N_2_ sorption isotherms and (**C**, **D**) pore size distribution of different MON-SH.


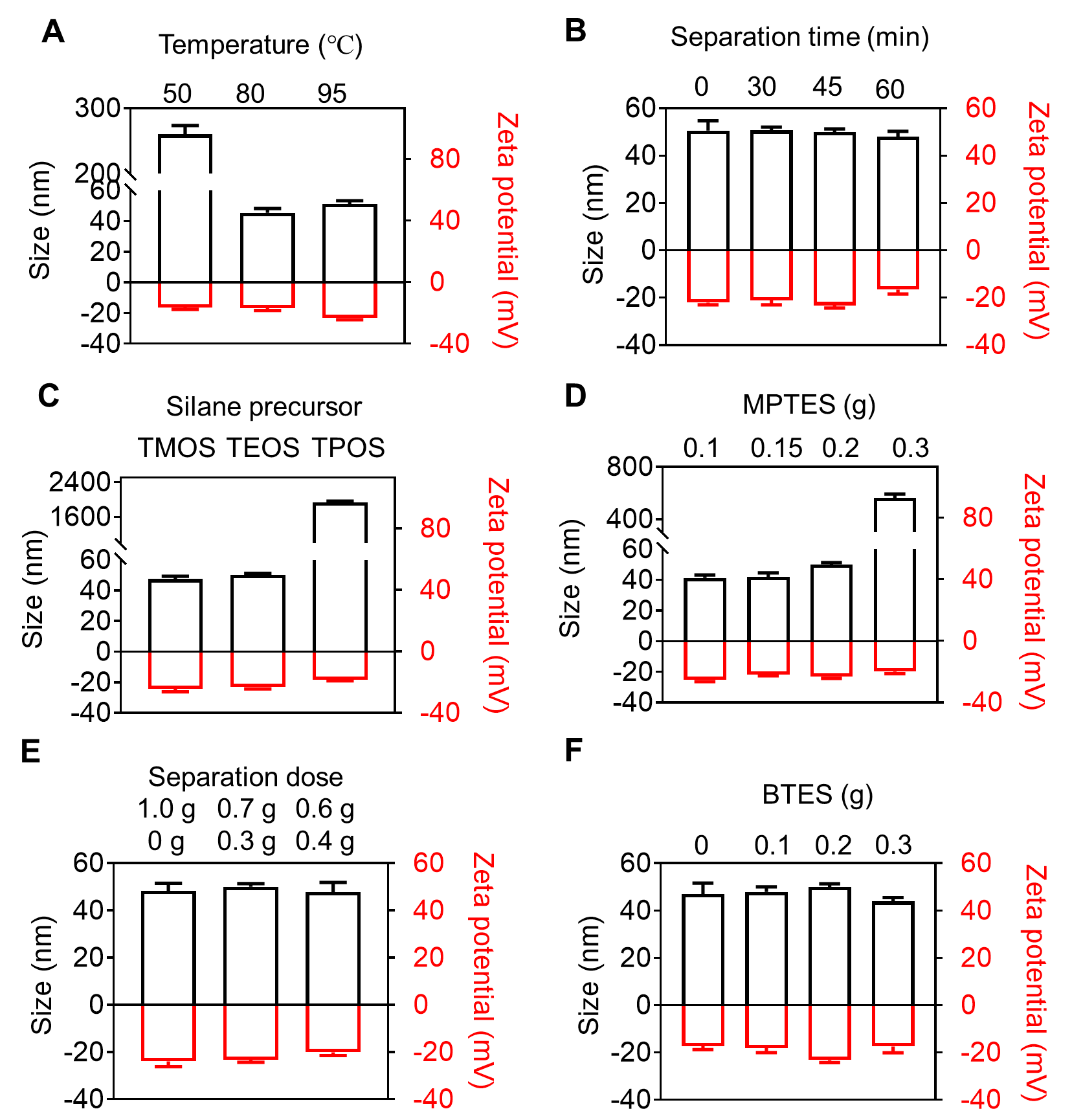


**Supplementary Fig. 4.** Hydrodynamic size and ζ-Potential of MON-SH.

**Supplementary Fig. 5.** The content of disulfide bond, active and inert thiol group in the framework of MON-SH by ICP-MS and Ellman’s reagent.


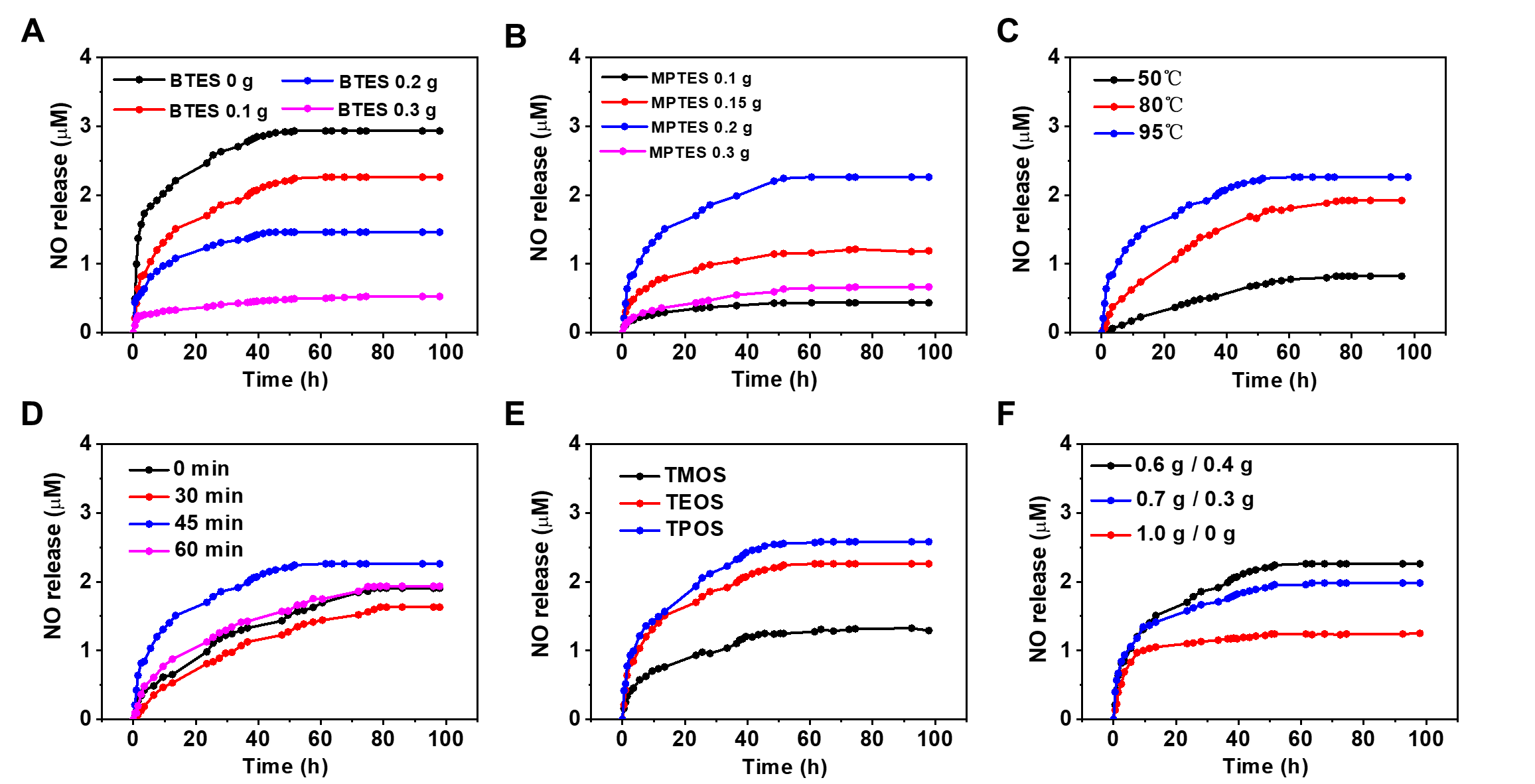


**Supplementary Fig. 6.** Cumulative NO release profiles of different MON-SNO.


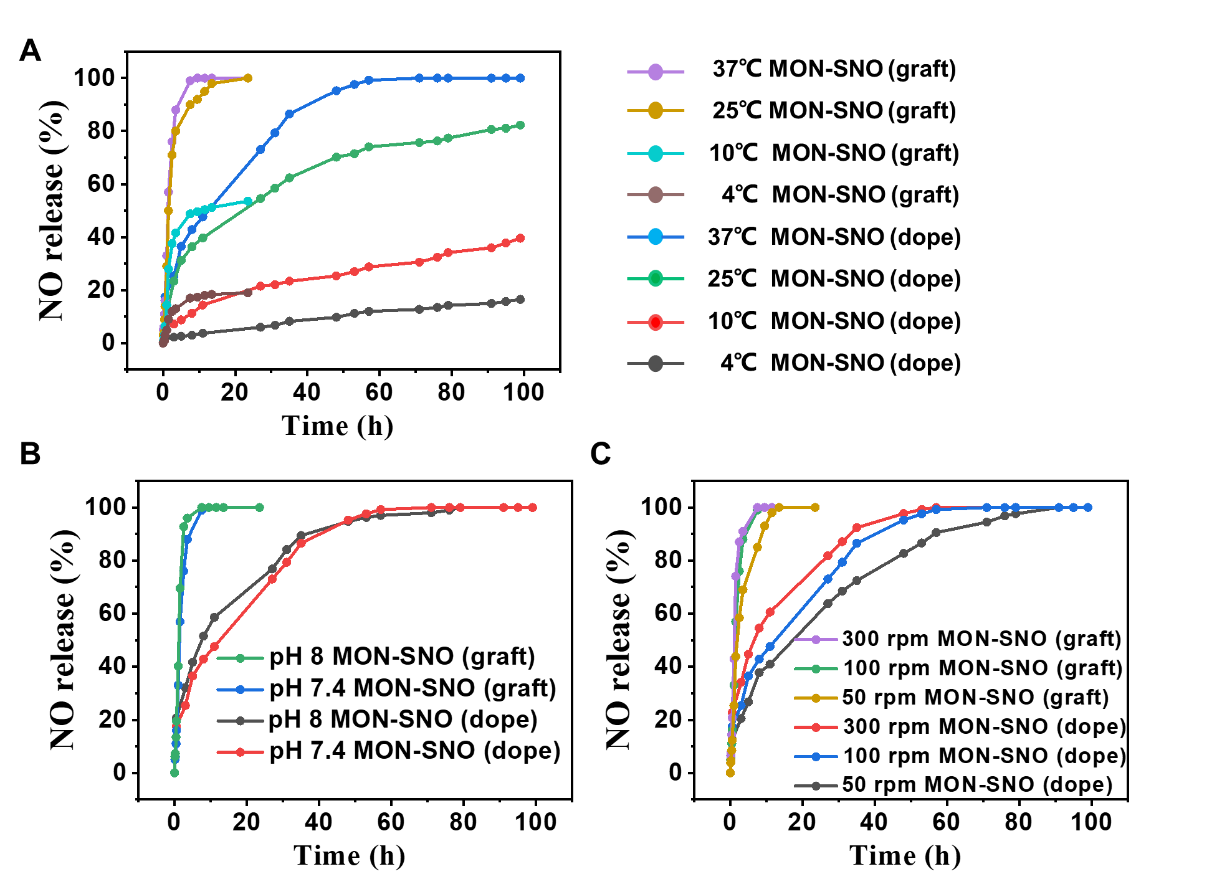


**Supplementary Fig. 7.** (**A** to **C**) Cumulative NO release profiles of MON-SNO (graft) and MON-SNO (dope) under different release conditions. (A) Temperature, (B) pH, (C) Shaking speed.


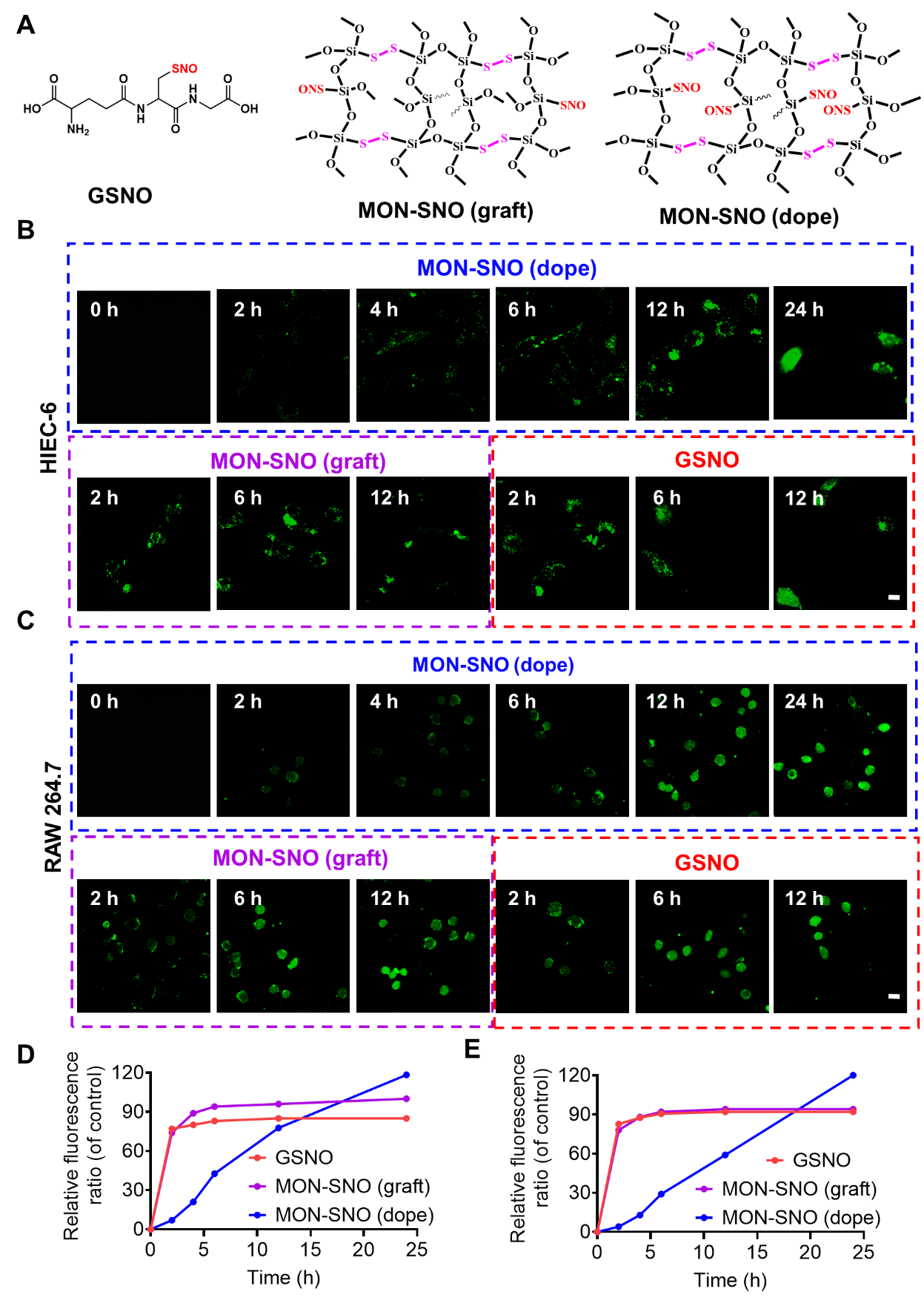


**Supplementary Fig. 8.** (**A**) Structure of GSNO, MON-SNO (graft) and MON-SNO (dope). (**B**) Cumulative NO release from GSNO, MON-SNO (graft) and MON-SNO (dope) in HIEC-6 cells. Scale bar: 20 μm. (**C**) Cumulative NO release from GSNO, MON-SNO (graft) and MON-SNO (dope) in RAW 264.7 macrophages. Scale bar: 20 μm. (**D** to **E**) Cumulative NO release from GSNO, MON-SNO (graft) and MON-SNO (dope) in (D) HIEC-6 cells and (E) RAW 264.7 macrophages.


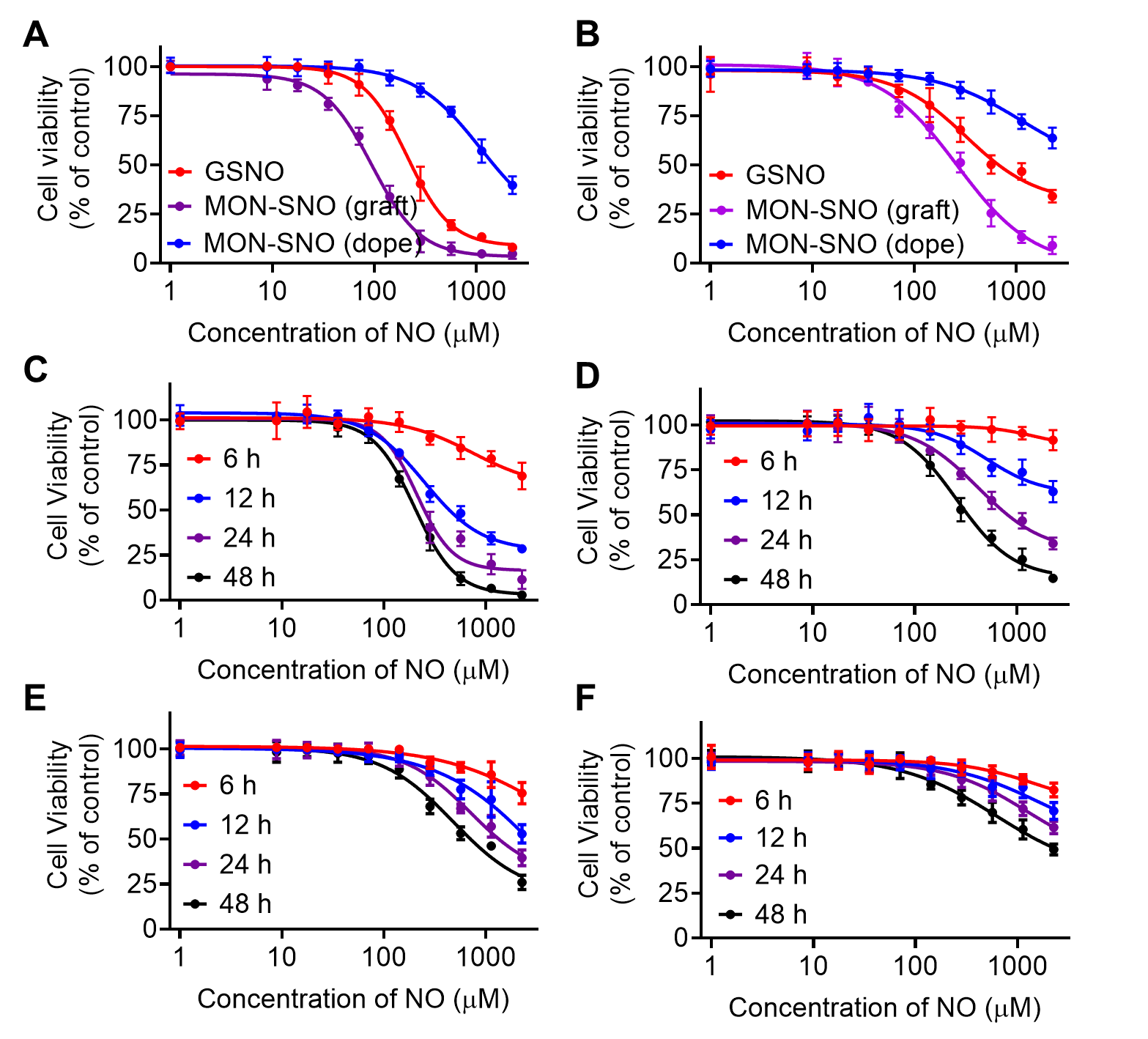


**Supplementary Fig. 9.** (**A** to **B**) The viability of (A) RAW 264.7 macrophages, (B) HIEC-6 cells incubated with MON-SNO (dope), MON-SNO (graft) and GSNO for 24 h via SRB assay. (**C** to **D**) The viability of (C) RAW 264.7 macrophages and (D) HIEC-6 cells incubated with GSNO for different times via SRB assay. (**E** to **F**) The viability of (E) RAW 264.7 macrophages and (F) HIEC-6 cells incubated with MON-SNO (dope) for different times via SRB assay.


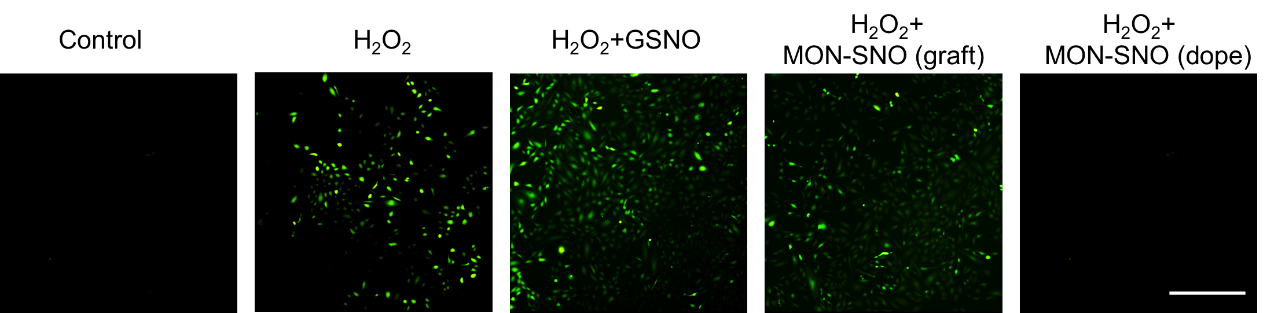


**Supplementary Fig. 10.** Fluorescent images of cellular ROS against HIEC-6 cells after treatment with different formulations. Scale bar: 100 µm.

*
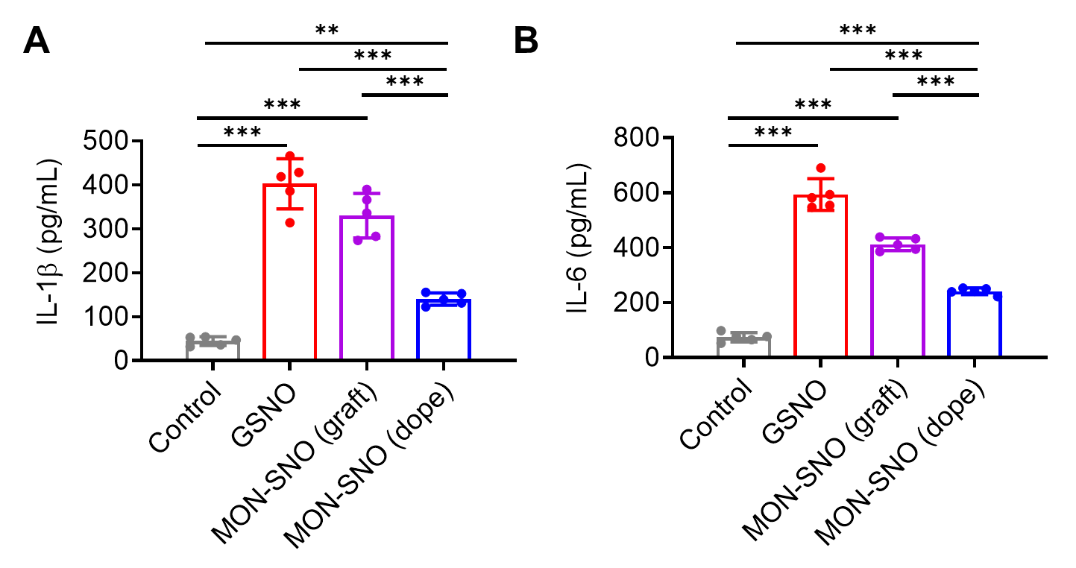
*

**Supplementary Fig. 11.** RAW 264.7 macrophages were stimulated with GSNO, MON-SNO (graft) and MON-SNO (dope). Supernatants were assayed for (A) IL-1β, (B) IL-6 by ELISA (n = 5). Data were presented as mean ± s.d. and the statistical significance was calculated via one-way ANOVA with Tukey’s multiple comparisons test. ***p* < 0.01, ****p* < 0.001.


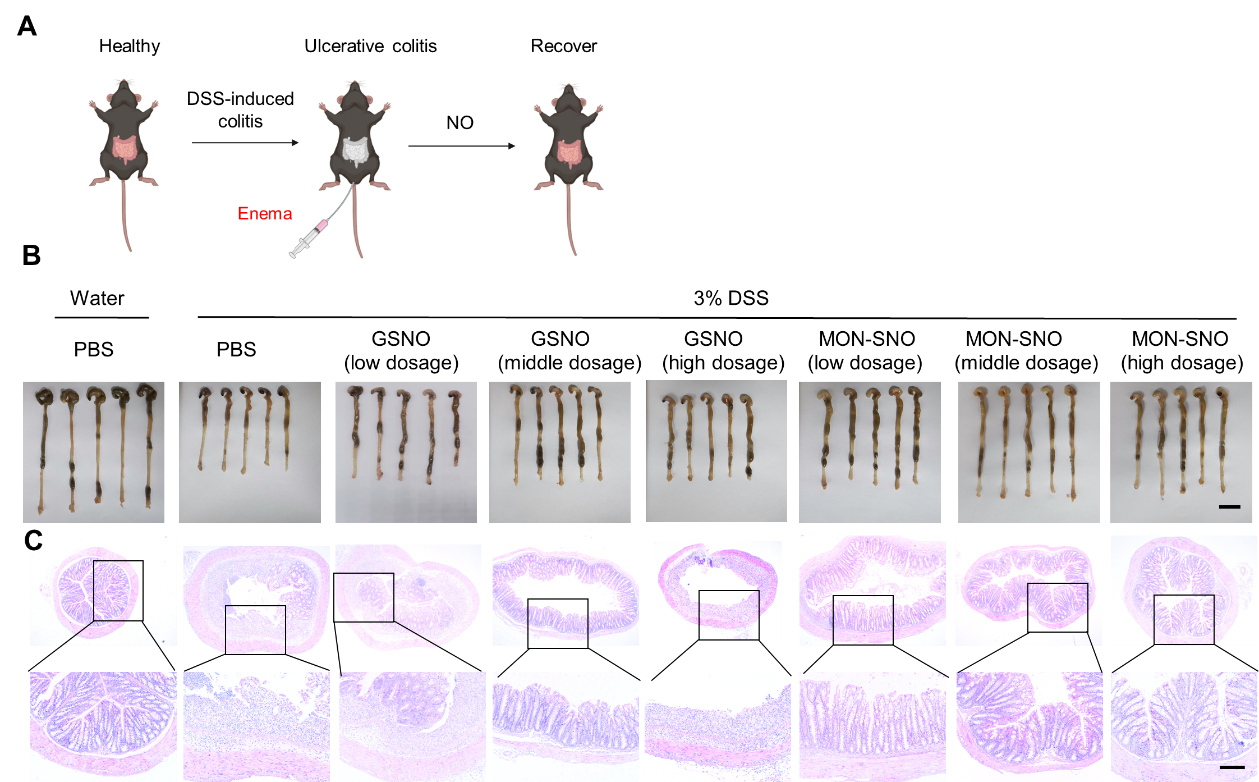


**Supplementary Fig. 12.** (**A**) NO demonstrates therapeutic efficacy in DSS-induced colitis of C57BL/6 mice. (**B**) Representative images of the colon in each group. Scale bar: 2 cm. (**C**) Representative images of colon sections of each group were stained with H&E. Scale bar: 100 μm.

**Supplementary Fig. 13.** Hydrodynamic size and ζ-Potential of the MON-SH, MON-SNO and MON-SNO@Dex.


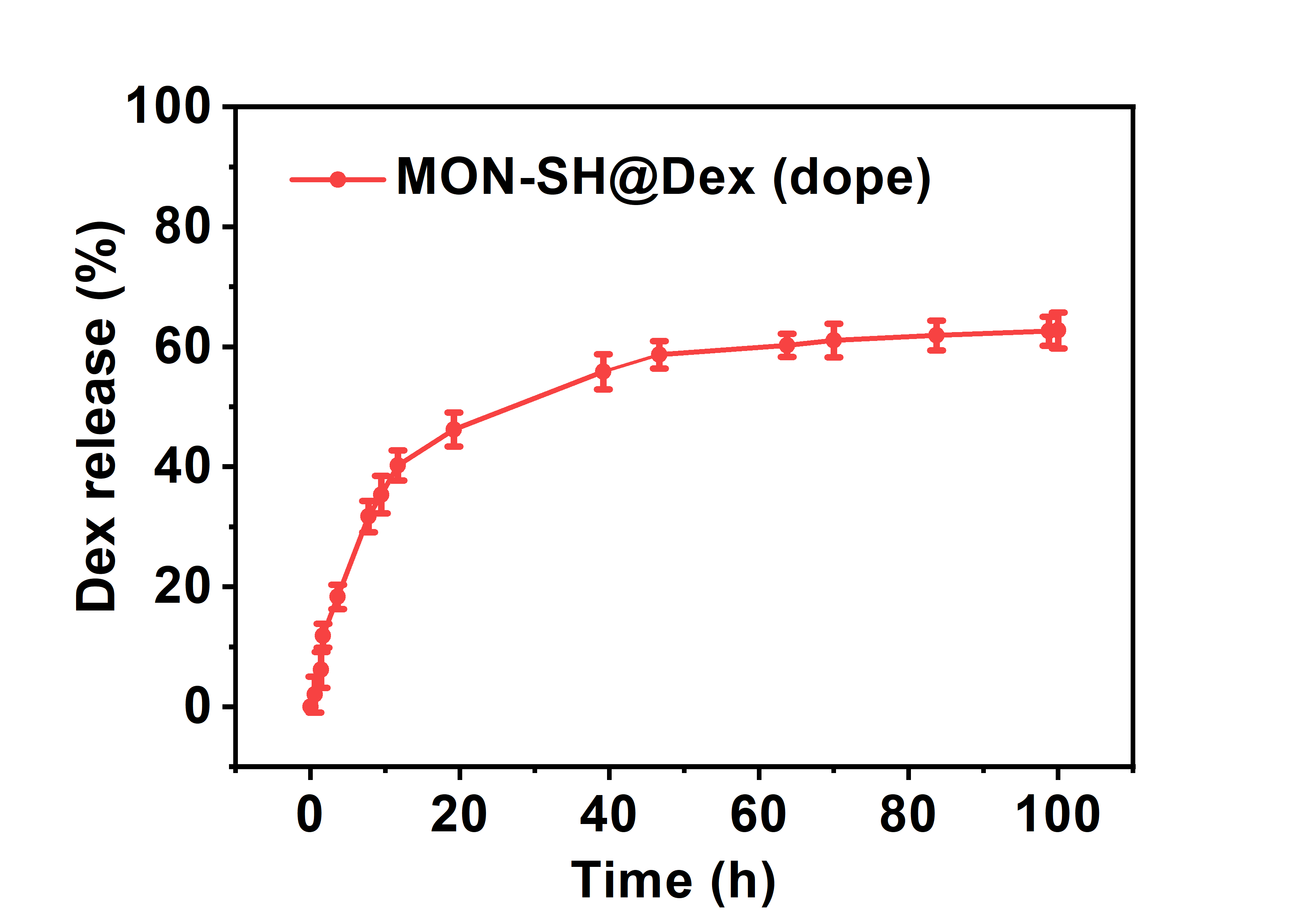


**Supplementary Fig. 14.** Cumulative Dex release profile of MON-SH@Dex (dope).


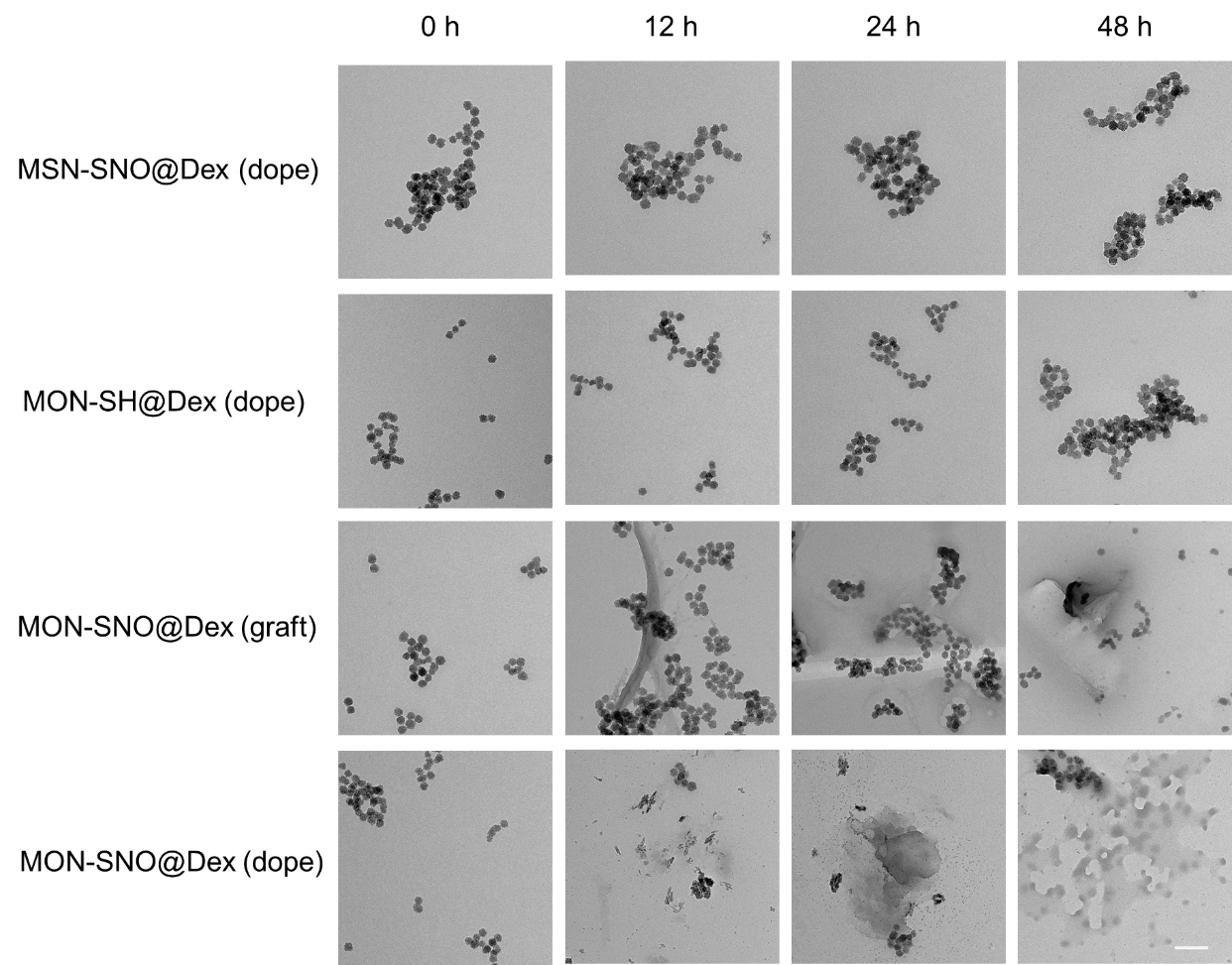


**Supplementary Fig. 15.** The degradation TEM images of MSN-SNO@Dex (dope), MON-SH@Dex (dope), MON-SNO@Dex (graft) and MON-SNO@Dex (dope) after incubation in SBF at different times. Scale bar: 200 μm.


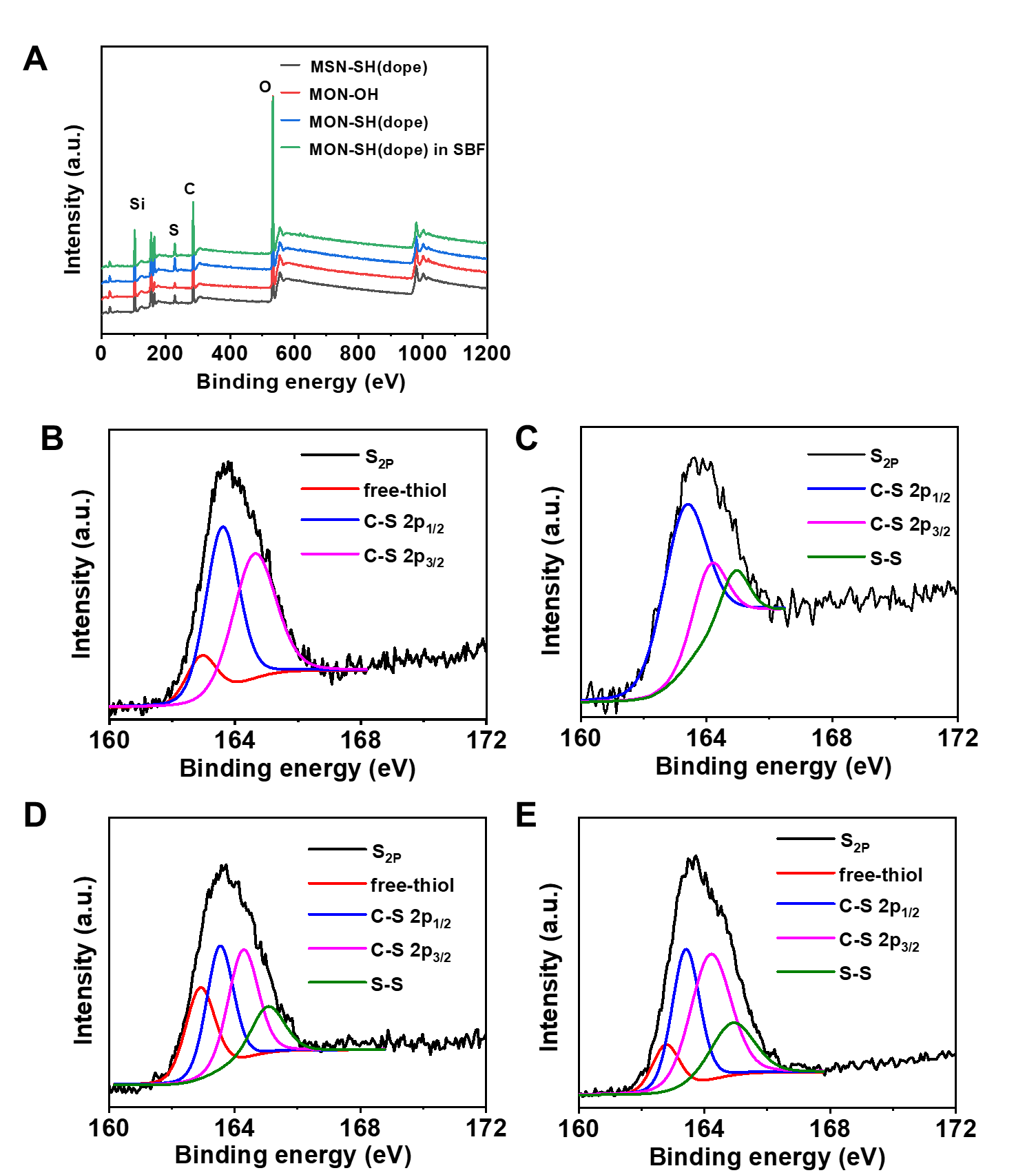


**Supplementary Fig. 16.** (**A**) XPS survey-wide scans of MSN-SH (dope), MON-OH; MON-SH (dope) before and after incubation in SBF solutions for 24 h. (**B** to **E**) S_2P_ of (B) MSN-SH (dope), (C) MON-OH; (D) MON-SH (dope). (E) S_2P_ of MON-SH (dope) after incubation in SBF solutions for 24 h. Free thiol (162.5 eV); C-S 2p_1/2_ (163.7 eV); C-S 2p_3/2_ (164.3 eV); S-S (165 eV) regions.

**Supplementary Fig. 17.** XPS survey-wide scans of MON-SNO (dope) after incubation in SBF solutions for 0, 12, 24 and 48 h.


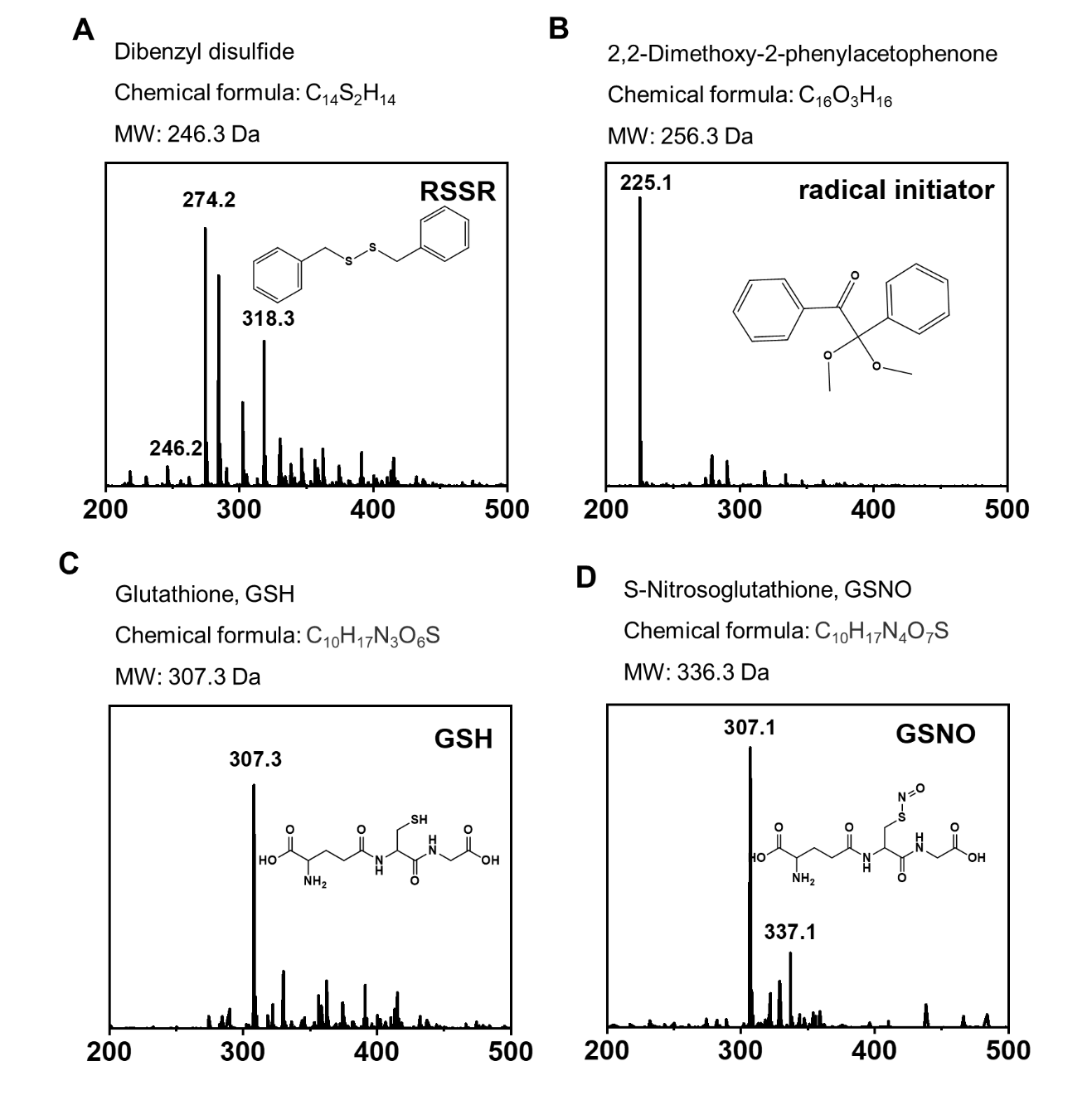


**Supplementary Fig. 18.** (**A**) ESI-MS of dibenzyl disulfide dissolved in methanol. (**B** to **D**) ESI-MS of (B) 2,2-Dimethoxy-2-phenylacetophenone, (C) GSH and (D) GSNO dissolved in water.


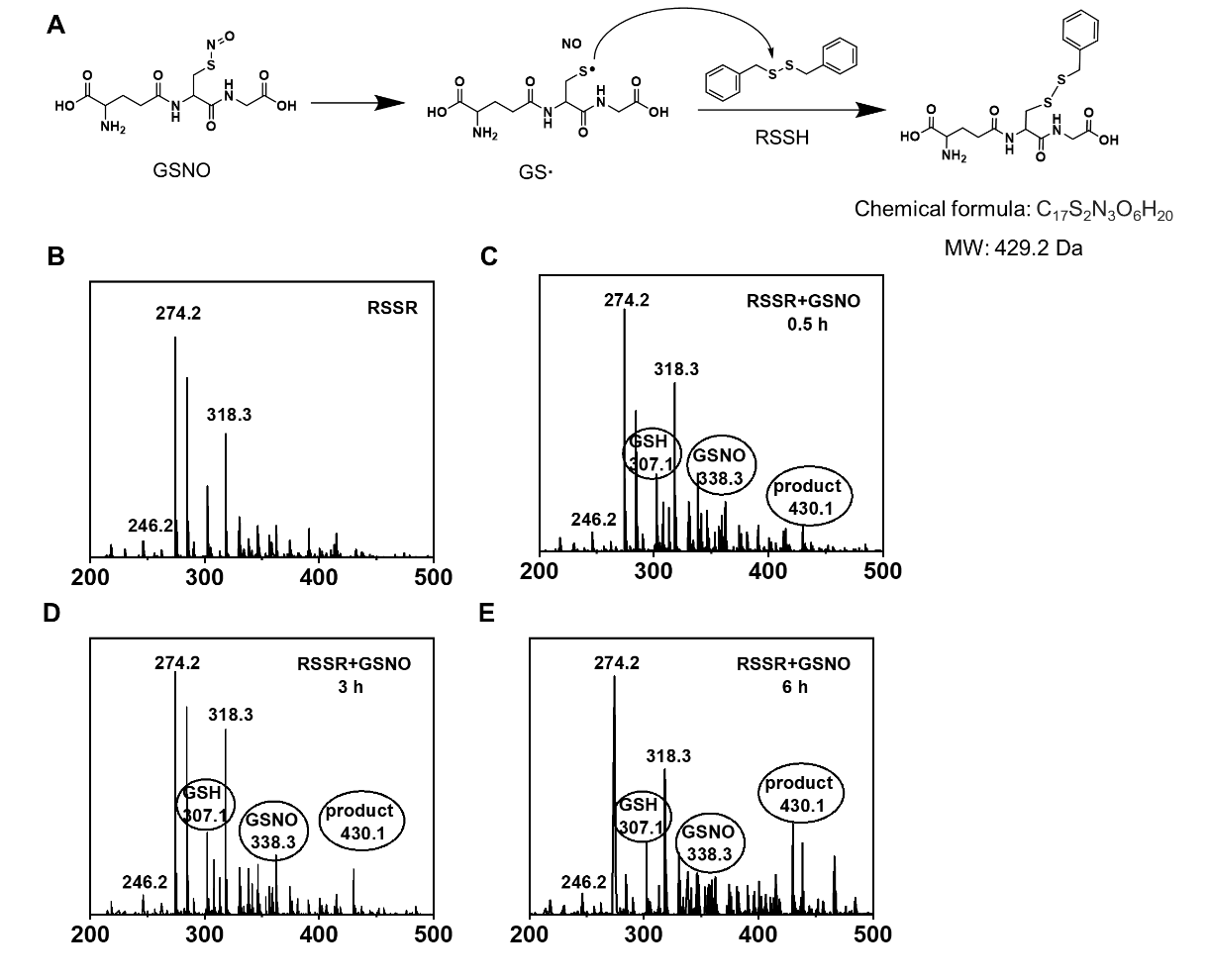


**Supplementary Fig. 19.** (**A**) Schematic of thiol–disulfide exchange. (**A**) ESI-MS of dibenzyl disulfide dissolved in methanol. (**C** to **E**) ESI-MS of the reaction product of dibenzyl disulfide (10 mM) and GSNO (2 mM) for (C) 0.5, (D) 3 and (E) 6 h.


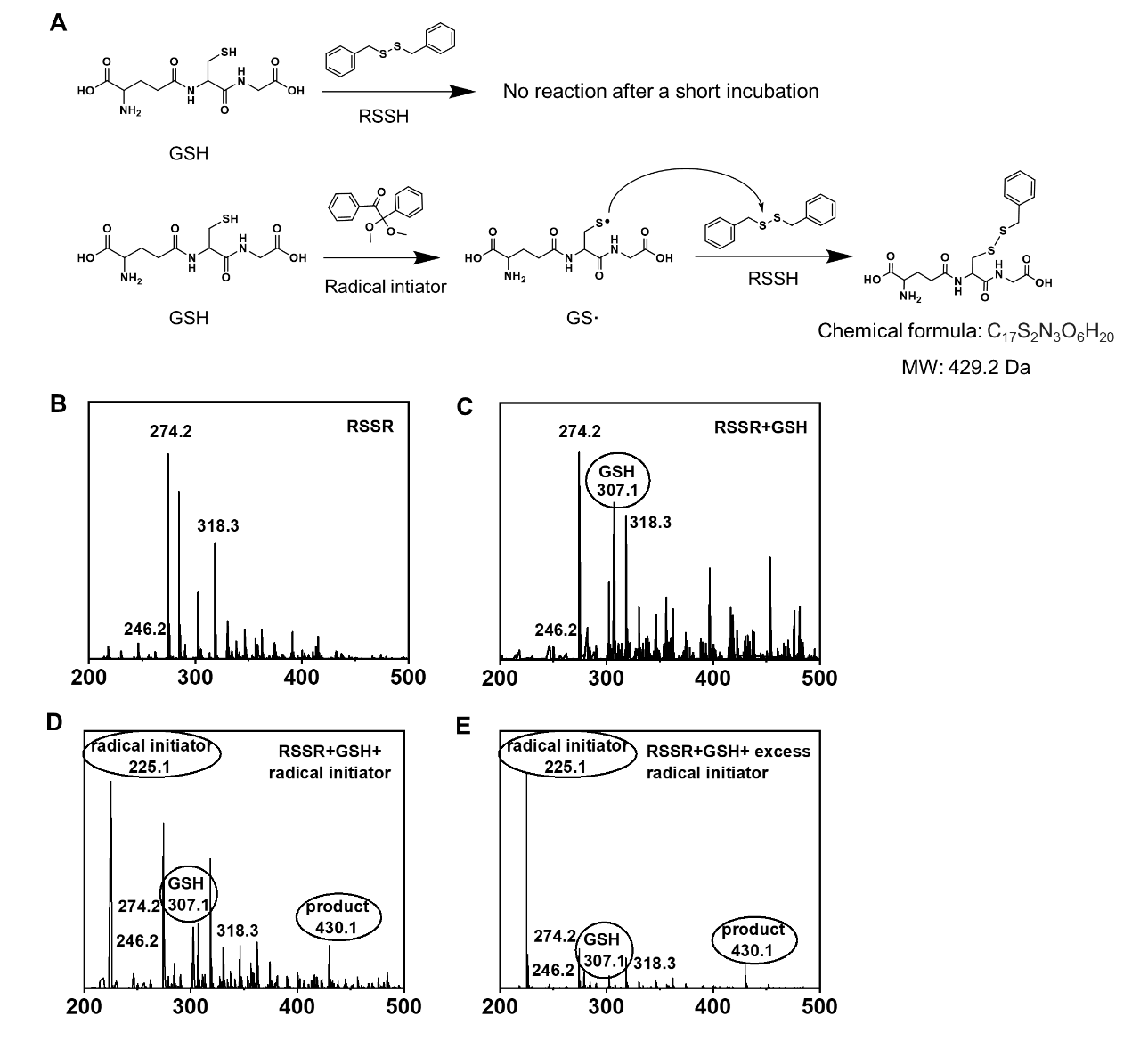


**Supplementary Fig. 20.** (**A**) Schematic of thiol–disulfide exchange. (**B**) ESI-MS of dibenzyl disulfide dissolved in methanol. (**C**) ESI-MS of the reaction product of dibenzyl disulfide (10 mM) and GSH (2 mM) for 6 h. (**D**) ESI-MS of the reaction product of dibenzyl disulfide (10 mM), GSH (2 mM) and 2,2-Dimethoxy-2-phenylacetophenone (10 mM) for 6 h. (**E**) ESI-MS of the reaction product of dibenzyl disulfide (10 mM), GSH (2 mM) and excess 2,2-Dimethoxy-2-phenylacetophenone (30 mM) for 6 h.


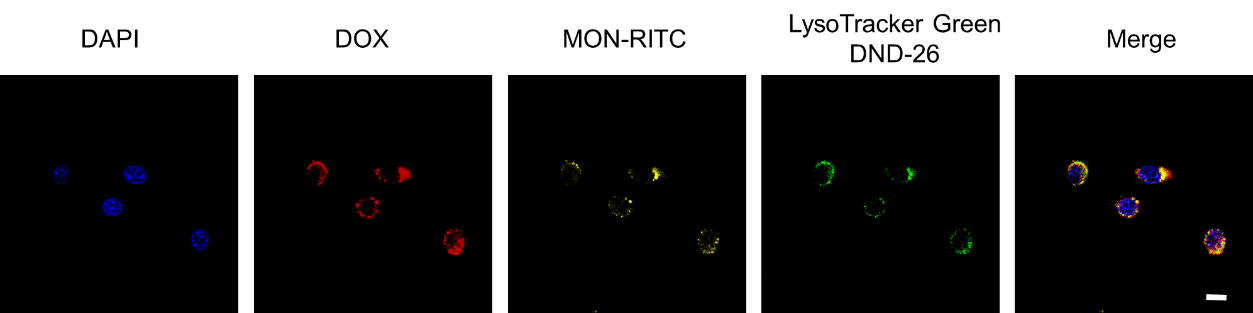


**Supplementary Fig. 21.** Fluorescent images of RAW 264.7 macrophages treated with MON-RITC@DOX for 6 h. DAPI and LysoTracker Green were used to stain cell nuclei (blue) and lysosomes (green), respectively. Scale bar: 20 μm.


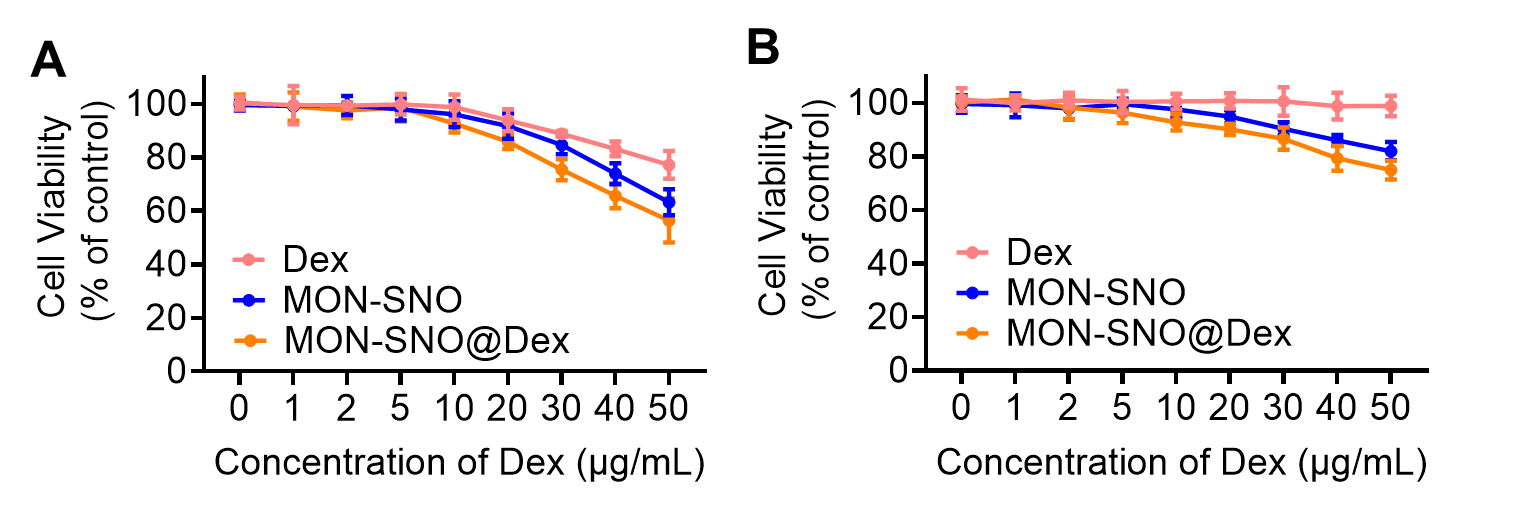


**Supplementary Fig. 22.** The viability of (**A**) RAW 264.7 macrophages and (**B**) HIEC-6 cells incubated with Dex, MON-SNO and MON-SNO@Dex for 24 h via SRB assay.


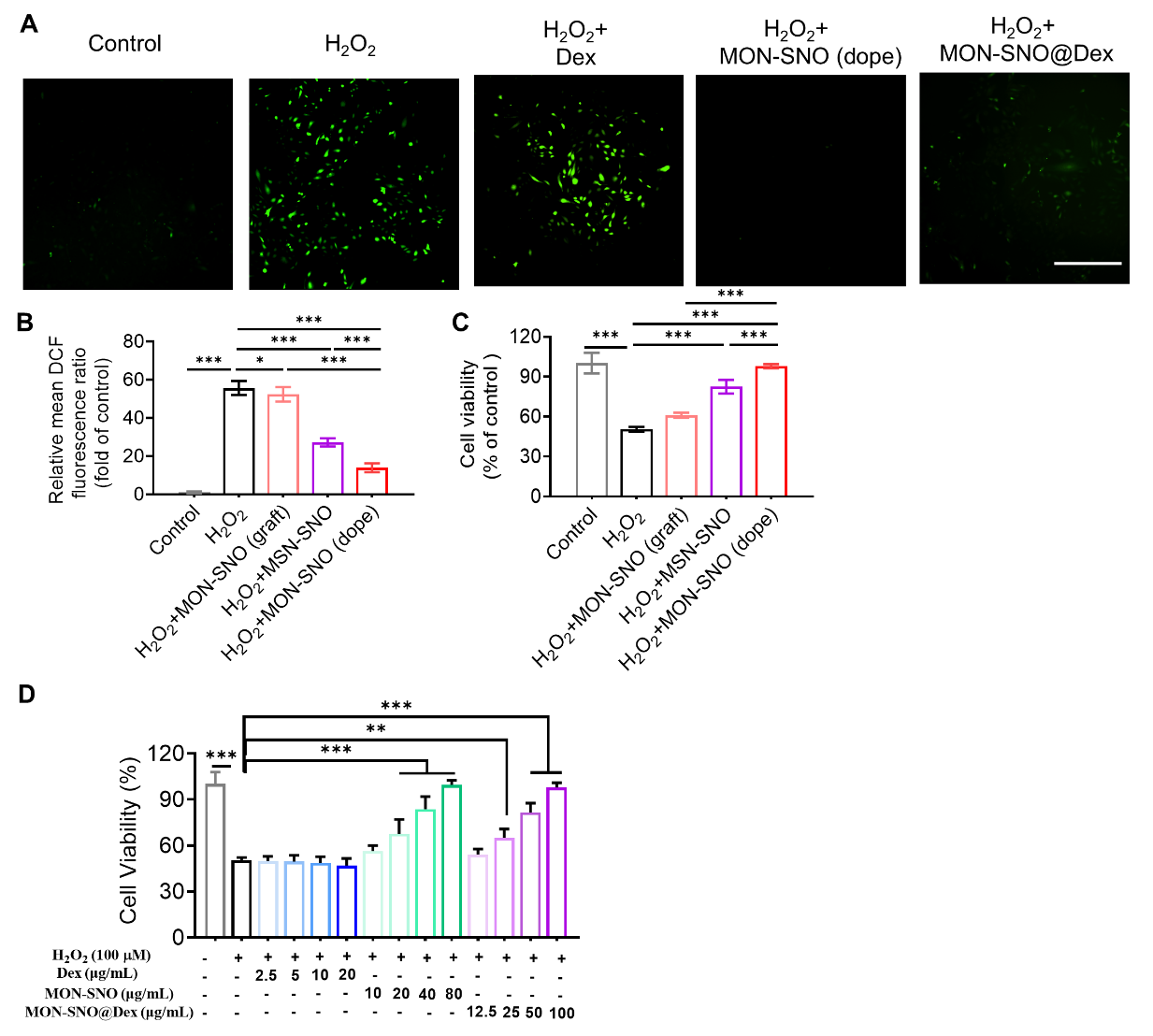


**Supplementary Fig. 23.** (**A**) Fluorescent images of cellular ROS against HIEC-6 cells after being treated with different formulations. Scale bar: 100 µm. (**B**) Relative fluorescence intensity of oxidized DCF in HIEC-6 cells after incubation with different formulations in the presence of H_2_O_2_ (100 µM). (**C** and **D**) The viability of HIEC-6 cells after incubation with different formulations in the presence of H_2_O_2_ (100 µM) (n = 5). Data were presented as mean ± s.d. and the statistical significance was calculated via one-way ANOVA with Tukey’s multiple comparisons test. **p* < 0.05, ***p* < 0.01, ****p* < 0.001.


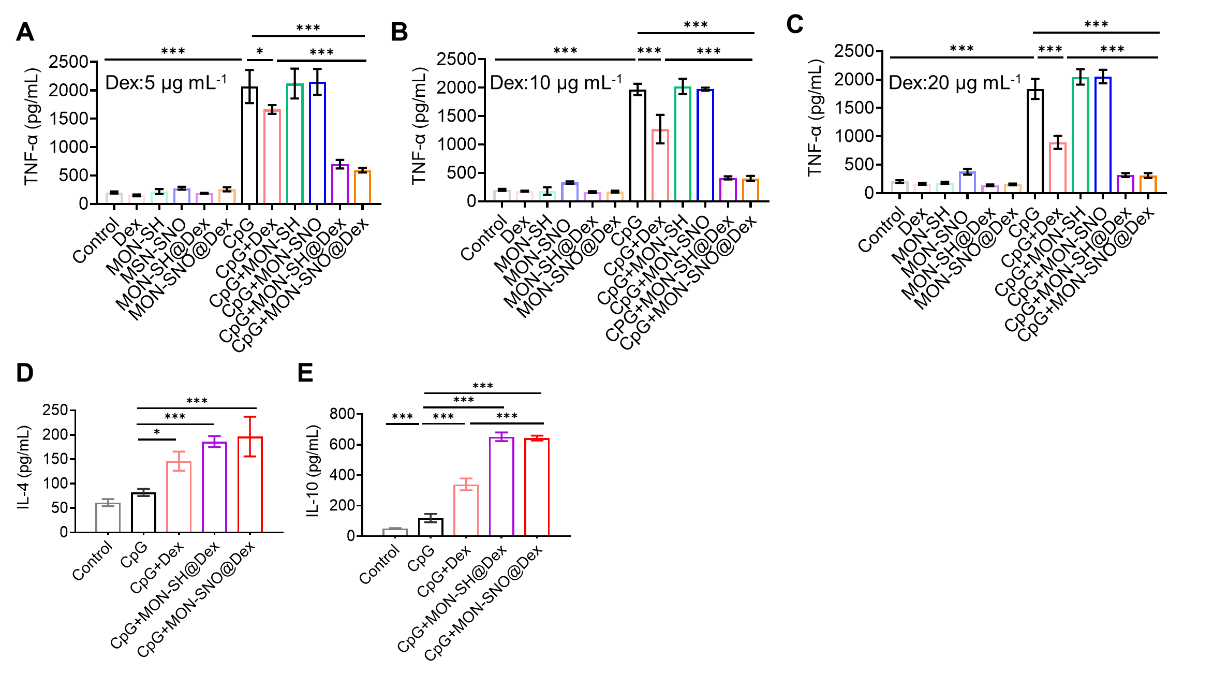


**Supplementary Fig. 24.** (**A** to **C**) RAW 264.7 macrophages were stimulated with different formulations in the absence or presence of CpG (1 μg mL^-1^). Supernatants were assayed for TNF-α by ELISA. (A) 5 µg mL^-1^ of Dex, 20 µg mL^-1^ of MON. (B) 10 µg mL^-1^ of Dex, 40 µg mL^-1^ of MON**.** (C) 20 µg mL^-1^ of Dex, 80 µg mL^-1^ of MON. (**D** to **G**) RAW 264.7 macrophages were stimulated with different formulations in the absence or presence of CpG (1 μg mL^-1^). (**D** to **E**) Supernatants were assayed for IL-4 (D) and IL-10 (E) by ELISA (n = 5). Data were presented as mean ± s.d. and the statistical significance was calculated via one-way ANOVA with Tukey’s multiple comparisons test. **p* < 0.05, ***p* < 0.01, ****p* < 0.001.

*
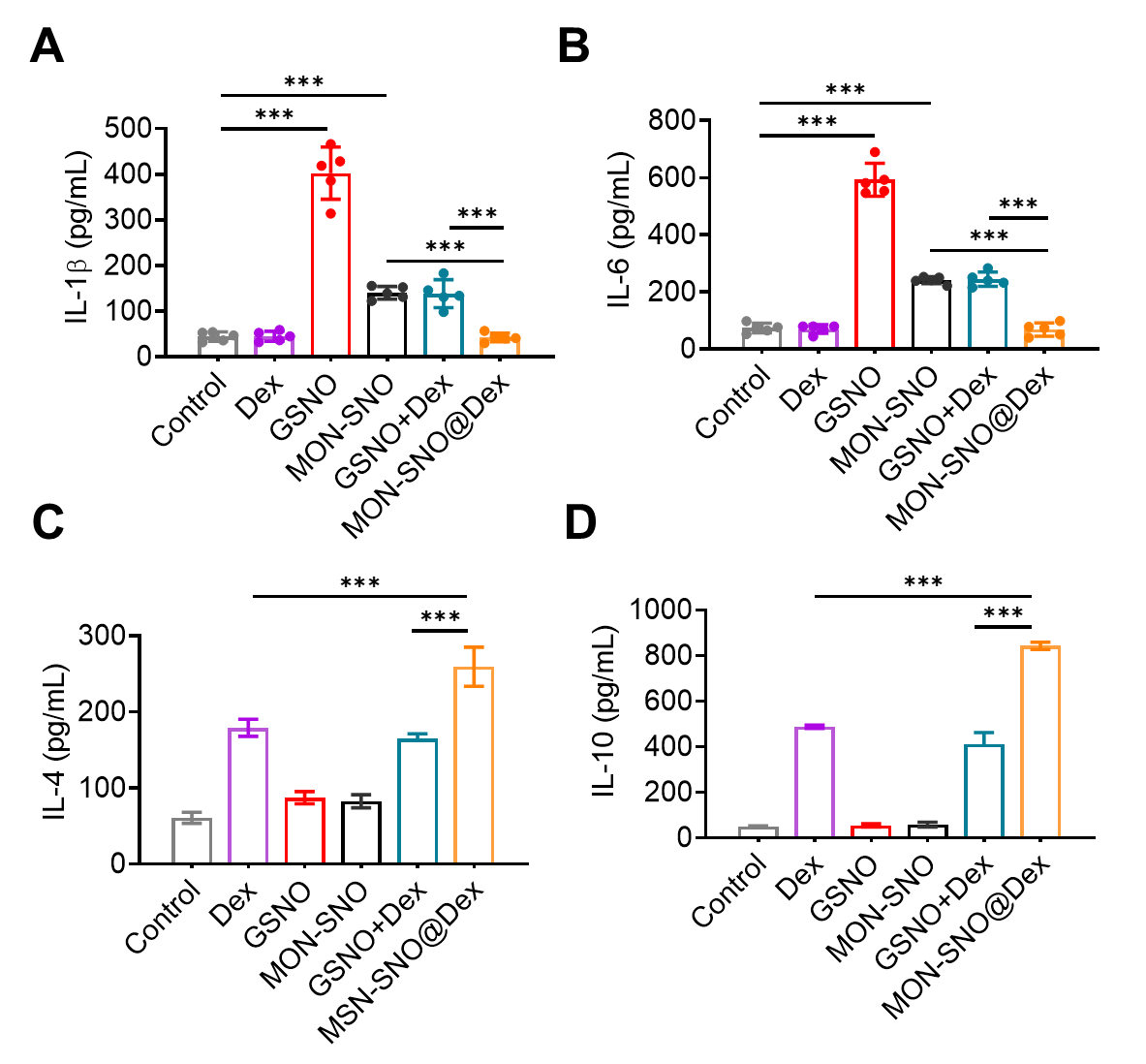
*

**Supplementary Fig. 25.** (**A** to **D**) RAW 264.7 macrophages were stimulated with MON-SNO, GSNO, Dex, GSNO+Dex and MON-SNO@Dex. Supernatants were assayed for (A) IL-1β, (B) IL-6, (C) IL-4 and (D) IL-10 by ELISA (n = 5). Data were presented as mean ± s.d. and the statistical significance was calculated via one-way ANOVA with Tukey’s multiple comparisons test. ****p* < 0.001.


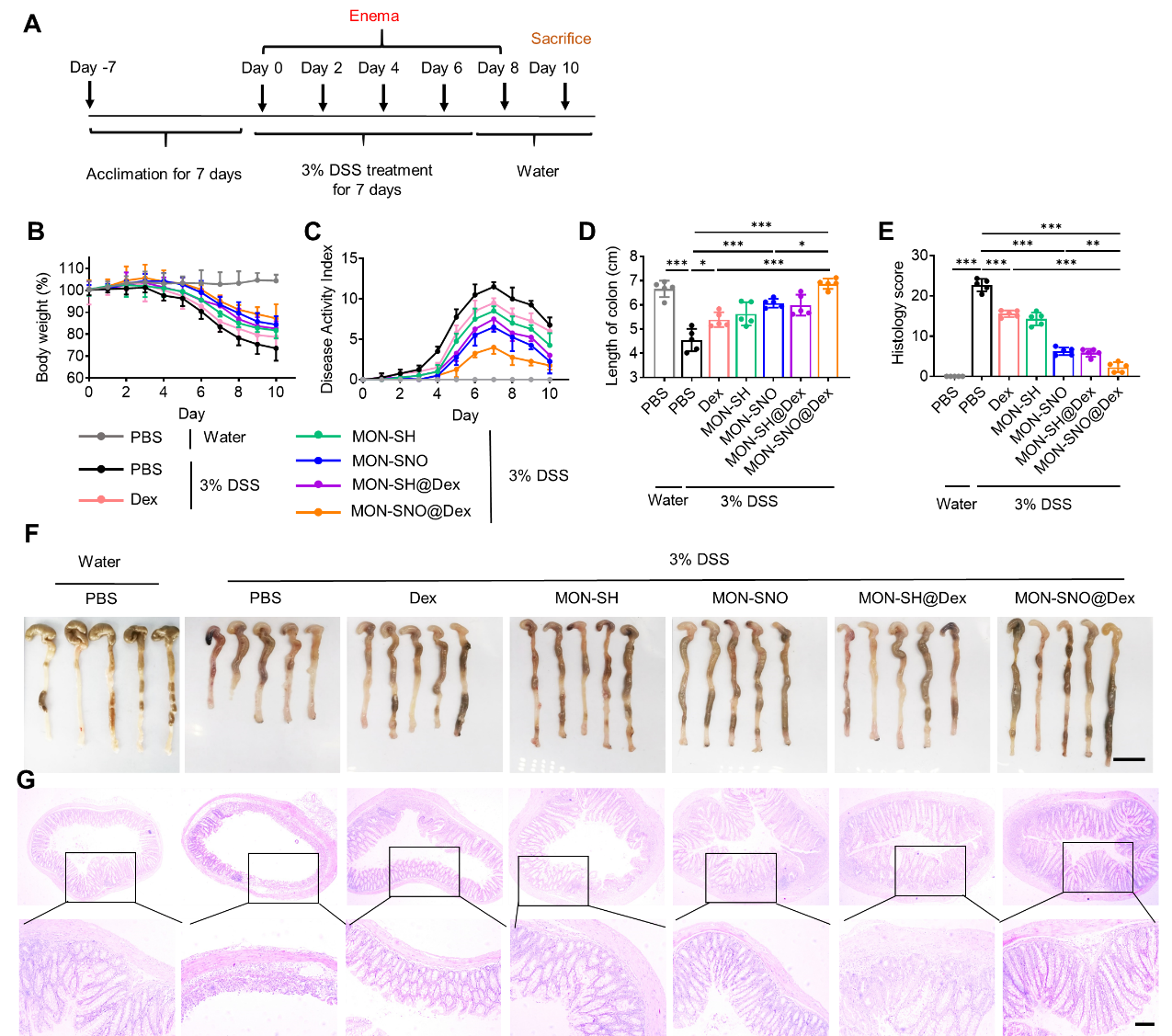


**Supplementary Fig. 26.** (**A**) C57BL/6 mice were provided with 3% DSS for 7 days. Rectal enema of different formulations on days 0, 2, 4, 6, and 8. (**B**) Daily body weight and (**C**) DAI changes of mice in each group for 10 days. (**D** to **E**) On day 10, mice were euthanized and (D) colon length, (E) colonic damage scores were measured (n = 5). (**F**) Representative images of the colon in each group. Scale bar: 2 cm. (**G**) Representative images of colon sections in each group were stained with H&E. Scale bar: 100 μm. Data were presented as mean ± s.d. and the statistical significance was calculated via one-way ANOVA with Tukey’s multiple comparisons test. **p* < 0.05, ***p* < 0.01, ****p* < 0.001.

**
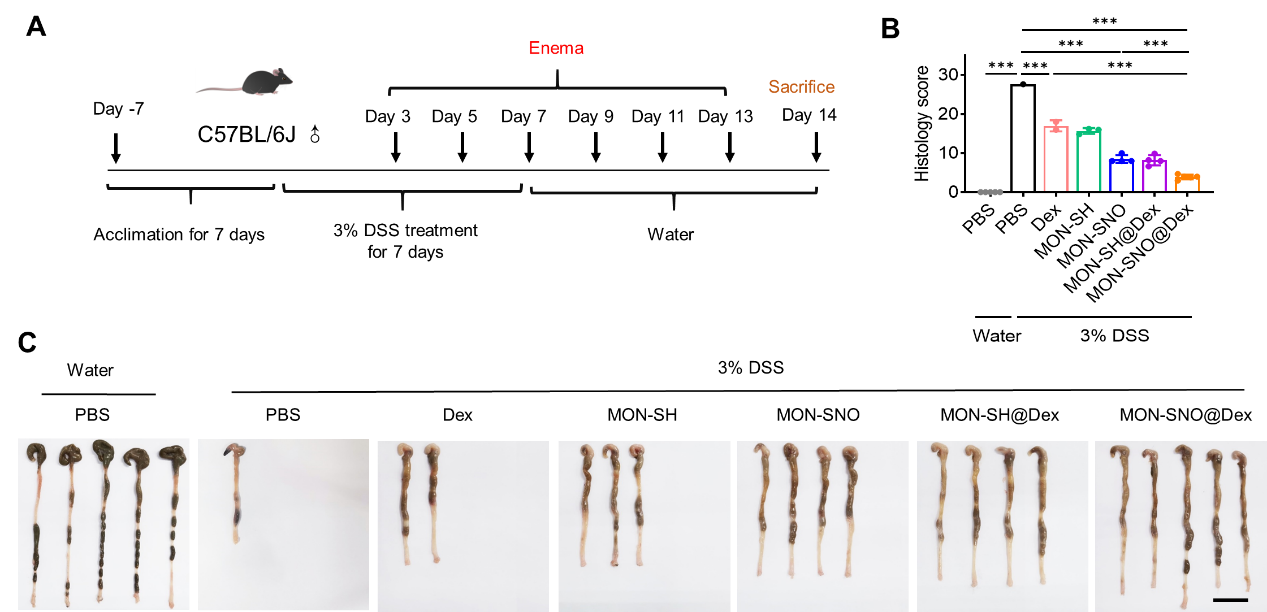
**

**Supplementary Fig. 27.** (**A**) C57BL/6 mice were provided with 3% DSS for 7 days. Rectal enema of different formulations on days 3, 5, 7, 9, 11 and 13. (**B**) On day 14, mice were euthanized and colonic damage scores were measured. (**C**) Representative images of the colon in each group. Scale bar: 2 cm. Data were presented as mean ± s.d. and the statistical significance was calculated via one-way ANOVA with Tukey’s multiple comparisons test. **p* < 0.05, ***p* < 0.01, ****p* < 0.001.


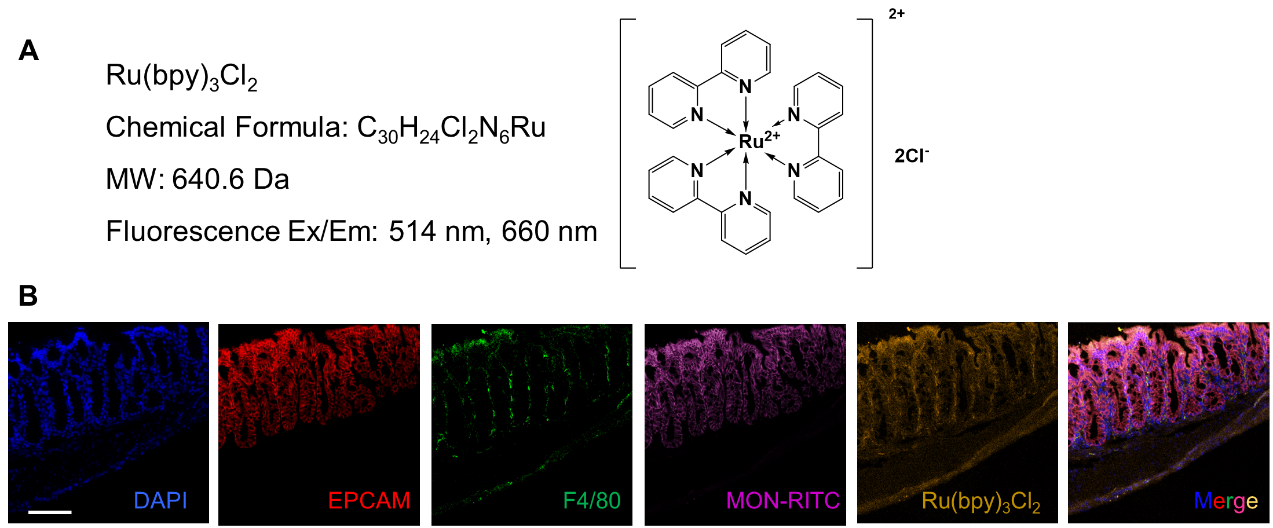


**Supplementary Fig. 28.** (**A**) Structure of Ru(bpy)_3_Cl_2_. (**B**) Fluorescent images of colon tissues, which were obtained from mice given 3% DSS water for 3 days and rectal enama administered with MON-RITC@Ru(bpy)_3_Cl_2_ (5 mg kg^-1^) (Blue: DAPI, red: EPCAM, green: macrophages, yellow: Ru(bpy)_3_Cl_2_, pink: MON). Scale bar: 100 μm.


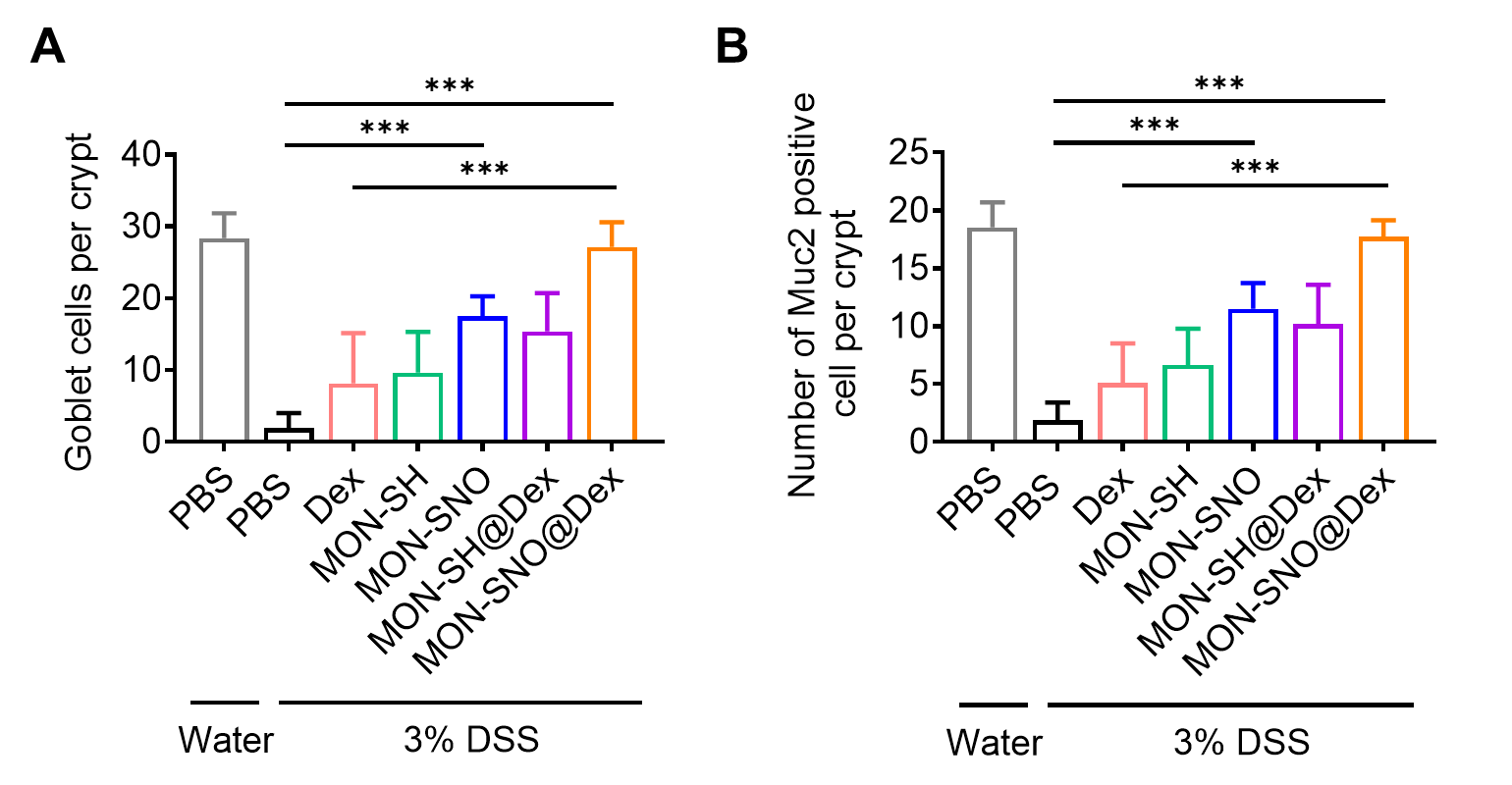


**Supplementary Fig. 29.** The quantification of goblet cell count and MUC2 protein in each crypt. Data were presented as mean ± s.d. and the statistical significance was calculated via one-way ANOVA with Tukey’s multiple comparisons test. ****p* < 0.001.


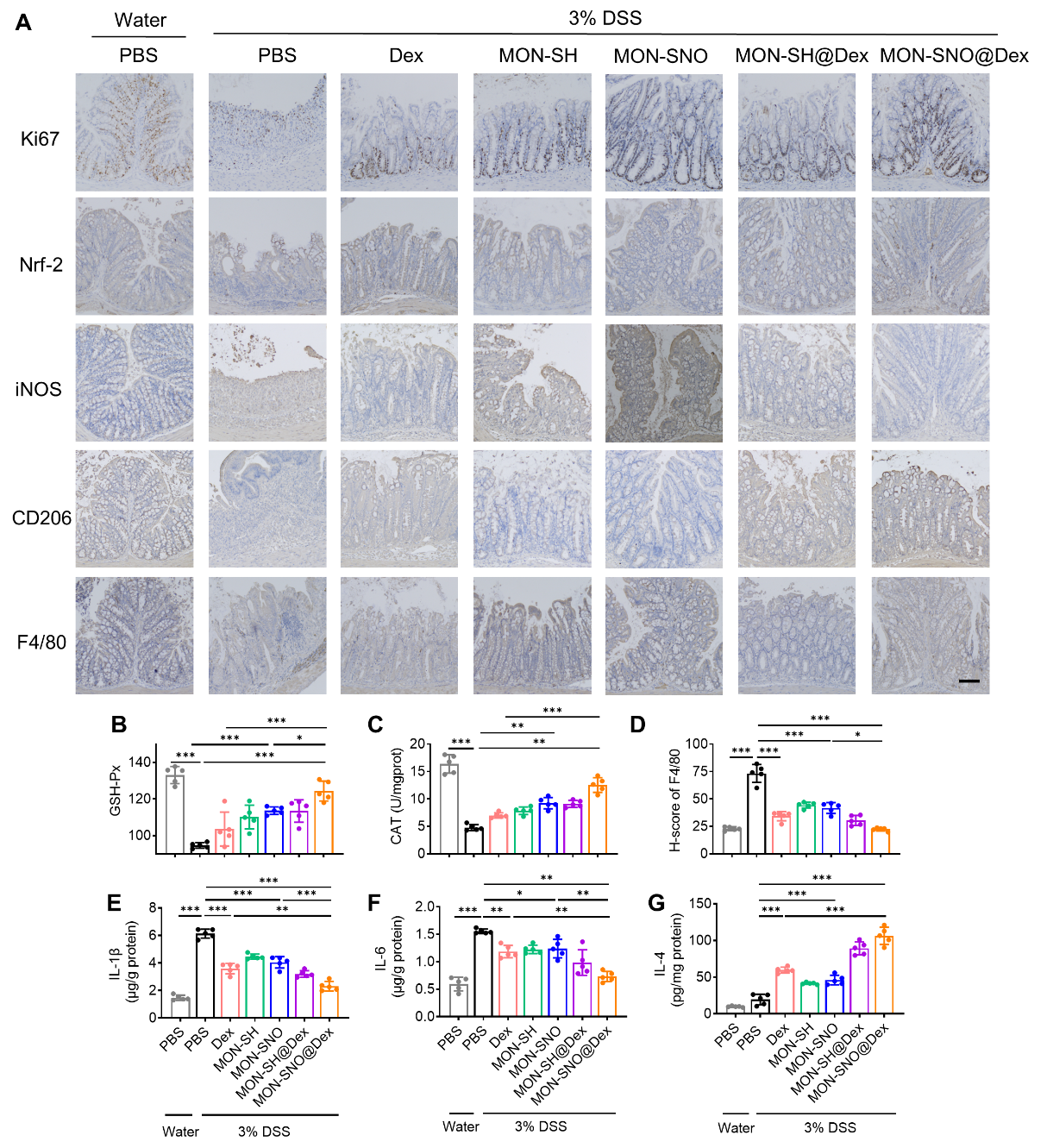


**Supplementary Fig. 30.** (**A)** Representative IHC images of Ki67, Nrf-2, iNOS, CD206, and F4/80 expression in colonic tissue sections of each group. Scale bars: 100 µm. (**B** to **C**) The colonic protein expression of (B) GSH-Px, (C) CAT from each group. (**D**) Quantification of F4/80 by immunohistochemical staining. (**E** to **G**) The colonic levels of cytokines (E) IL-1β, (F) IL-6 and (G) IL-4 (n = 5). Data were presented as mean ± s.d. and the statistical significance was calculated via one-way ANOVA with Tukey’s multiple comparisons test. **p* < 0.05, ***p* < 0.01, ****p* < 0.001.


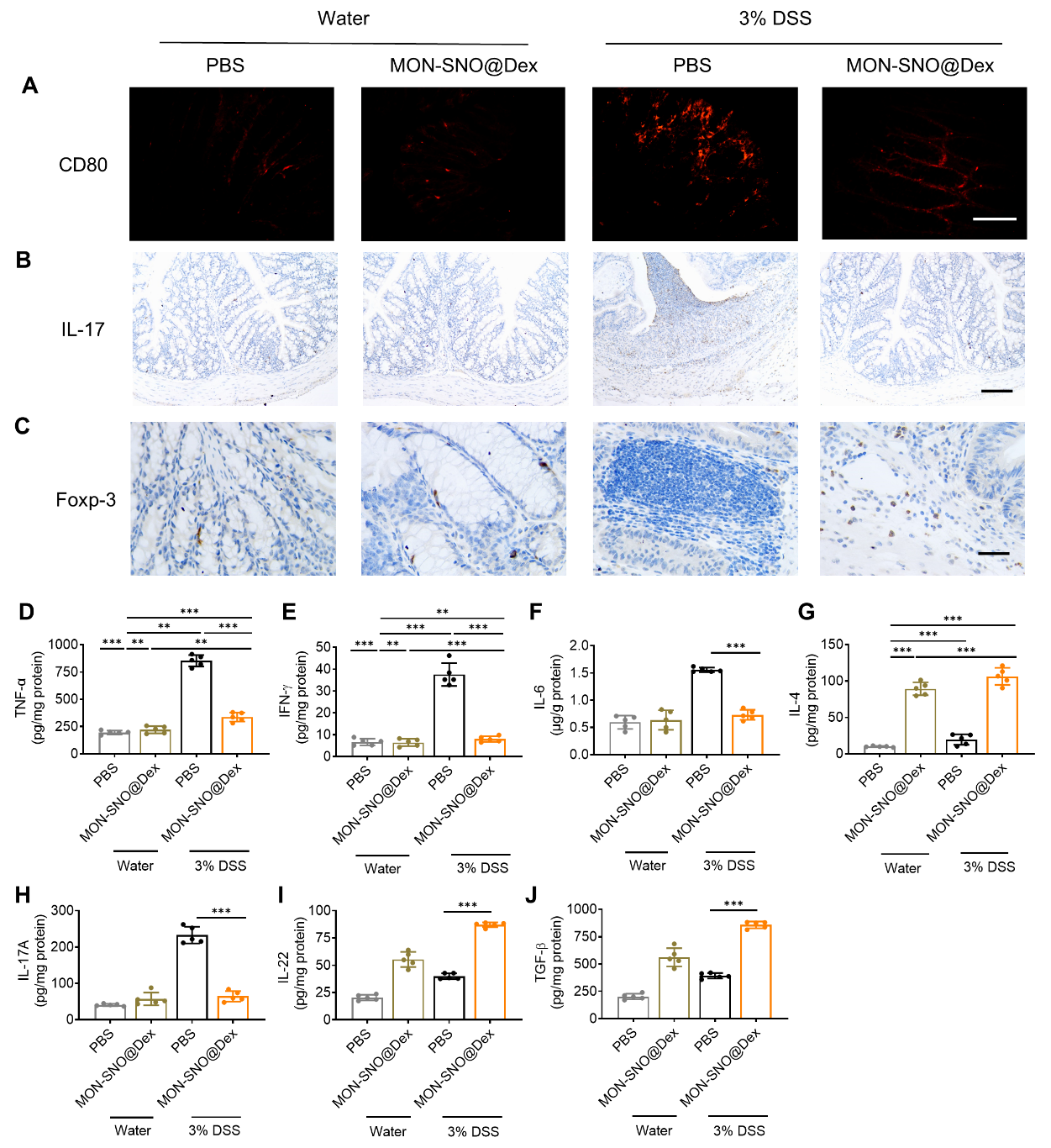


**Supplementary Fig. 31.** (**A)** Representative fluorescent images of CD80 expression in colonic sections of each group. Scale bars: 100 µm. (**B)** Representative IHC images of IL-17 expression in colonic sections of each group. Scale bars: 100 µm. (**C)** Representative IHC images of FoxP-3 expression in colonic sections of each group. Scale bars: 25 µm. (**D** to **J**) The colonic levels of cytokines (D) TNF-α, (E) IFN-γ, (F) IL-6, (G) IL-4, (H) IL-17A, (I) IL-22 and (J) TGF-β (n = 5). Data were presented as mean ± s.d. and the statistical significance was calculated via one-way ANOVA with Tukey’s multiple comparisons test. ***p* < 0.01, ****p* < 0.001.


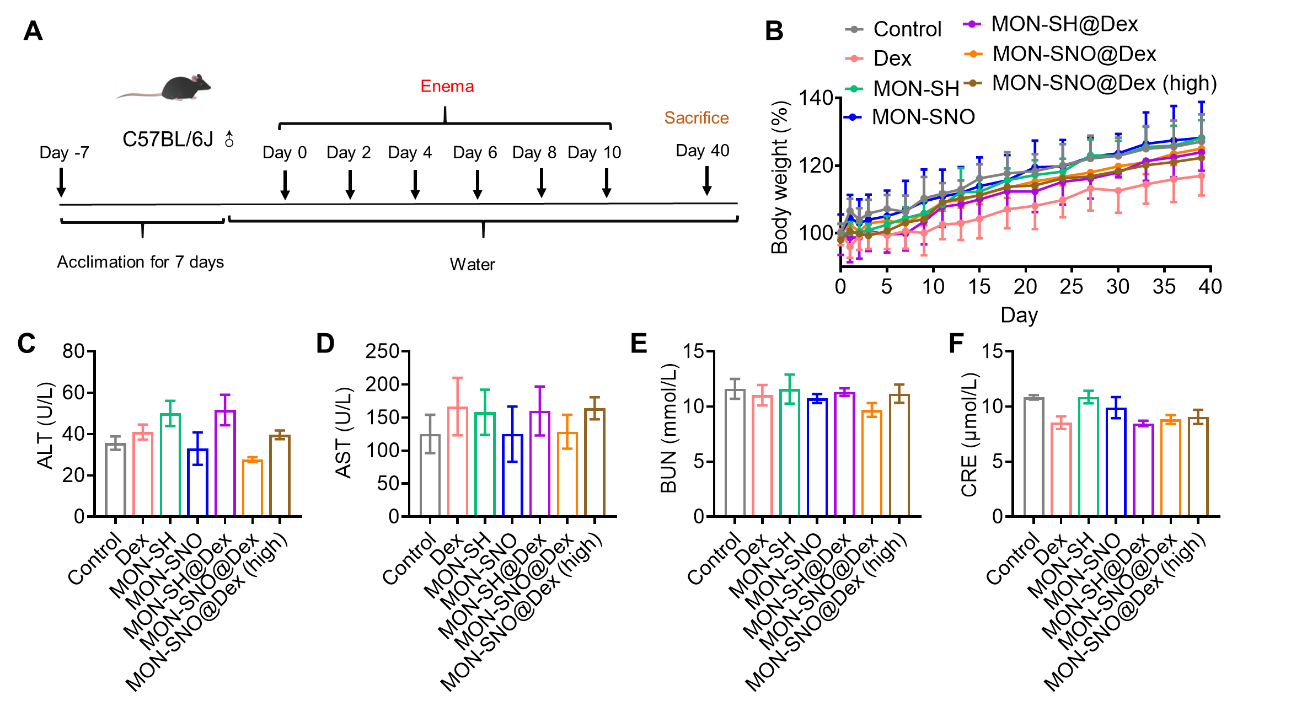


**Supplementary Fig. 32.** (**A**) The healthy C57BL/6 mice were administered a rectal enema of different formulations on days 0, 2, 4, 6, 8, 10. (**B**) Daily body weight of mice in each group for 40 days. (**C** to **F**) Serum biochemistry indicators of healthy mice treated with the different formulations, (C) ALT, (D) AST, (E) BUN and (F) CRE. Data were presented as mean ± SD (n = 5).


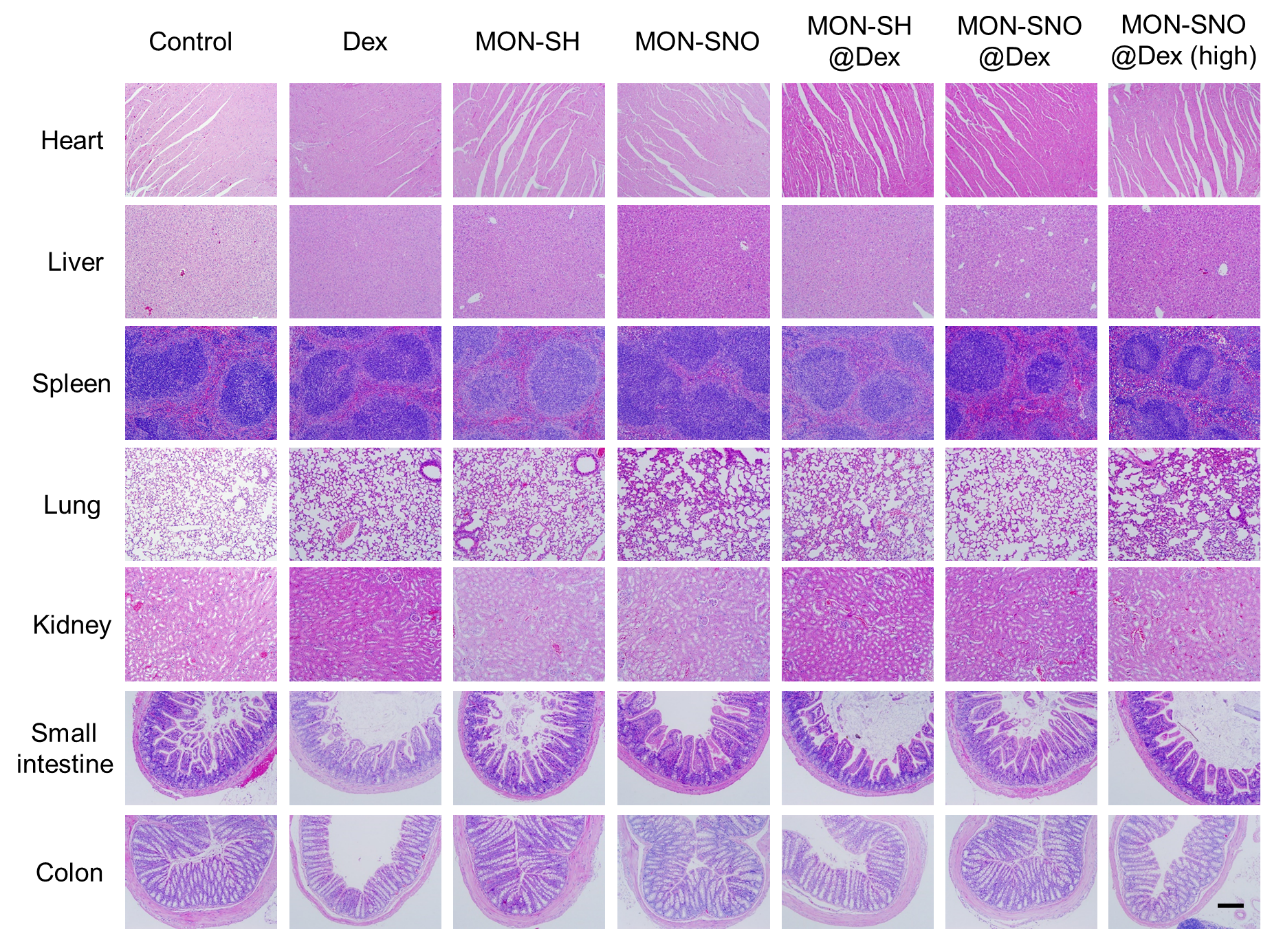


**Supplementary Fig. 33.** Representative images of H&E staining of heart, liver, spleen, lung and kidney, small intestine and colon of different groups in biosafety experiment. Scale bars: 100 µm.

**Supplementary Table 1** Summary of synthetic parameters and corresponding capacities with different MON-SNO (dope).

|  | | Silane  precursor | | Separation  dose (g) | Time  (min) | Temperat-ure(℃) | BTES(g) | MPTES (g) | | Size (nm) | Zeta (mV) | Thiol content  (μmol mg^-1^) | | NO content  (μmol mg^-1^) | NO release  time ~(h) |
| --- | --- | --- | --- | --- | --- | --- | --- | --- | --- | --- | --- | --- | --- | --- | --- |
|  | TEOS | | 1.0 (0.7/0.3) | | 45 | 50 | 0.1 | 0.2 | N/A | | -16.4±1.2 | 1.17 | 0.82 | | 70 |
|  | TEOS | | 1.0 (0.7/0.3) | | 45 | 80 | 0.1 | 0.2 | 45.4±2.8 | | -16.7±1.6 | 2.64 | 1.92 | | 70 |
|  | TEOS | | 1.0 (0.7/0.3) | | 45 | 95 | 0.1 | 0.2 | 51.2±2.1 | | -23.2±1.1 | 5.48 | 2.26 | | 60 |
|  | TMOS | | 1.0 (0.7/0.3) | | 45 | 95 | 0.1 | 0.2 | 46.9±1.4 | | -24.3±2.0 | 2.45 | 1.29 | | 60 |
|  | TEOS | | 1.0 (0.7/0.3) | | 45 | 95 | 0.1 | 0.2 | 51.2±2.1 | | -23.2±1.1 | 5.48 | 2.26 | | 60 |
|  | TPOS | | 1.0 (0.7/0.3) | | 45 | 95 | 0.1 | 0.2 | N/A | | -18.3±0.6 | 6.82 | 2.58 | | 60 |
|  | TEOS | | 1.0 (1.0/0) | | 45 | 95 | 0.1 | 0.2 | 47.7±3.1 | | -23.9±2.2 | 2.48 | 1.25 | | 60 |
|  | TEOS | | 1.0 (0.7/0.3) | | 45 | 95 | 0.1 | 0.2 | 51.2±2.1 | | -23.2±1.1 | 5.48 | 2.26 | | 60 |
|  | TEOS | | 1.0 (0.6/0.4) | | 45 | 95 | 0.1 | 0.2 | 47.6±4.6 | | -20.1±1.4 | 3.45 | 1.98 | | 60 |
|  | TEOS | | 1.0 (0.7/0.3) | | 0 | 95 | 0.1 | 0.2 | 50.1±4.4 | | -22.1±1.3 | 2.51 | 1.63 | | 70 |
|  | TEOS | | 1.0 (0.7/0.3) | | 30 | 95 | 0.1 | 0.2 | 50.6±1.4 | | -21.1±1.9 | 3.21 | 1.91 | | 70 |
|  | TEOS | | 1.0 (0.7/0.3) | | 45 | 95 | 0.1 | 0.2 | 51.2±2.1 | | -23.2±1.1 | 5.48 | 2.26 | | 60 |
|  | TEOS | | 1.0 (0.7/0.3) | | 60 | 95 | 0.1 | 0.2 | 48.0±2.2 | | -16.6±1.7 | 3.22 | 1.93 | | 70 |
|  | TEOS | | 1.0 (0.7/0.3) | | 45 | 95 | 0.1 | 0.1 | 41.3±2.1 | | -25.4±0.96 | 0.54 | 0.42 | | 60 |
|  | TEOS | | 1.0 (0.7/0.3) | | 45 | 95 | 0.1 | 0.15 | 41.5±2.8 | | -21.8±0.76 | 2.07 | 1.17 | | 60 |
|  | TEOS | | 1.0 (0.7/0.3) | | 45 | 95 | 0.1 | 0.2 | 51.2±2.1 | | -23.2±1.1 | 5.48 | 2.26 | | 60 |
|  | TEOS | | 1.0 (0.7/0.3) | | 45 | 95 | 0.1 | 0.3 | N/A | | -19.8±1.5 | 0.76 | 0.66 | | 60 |
|  | TEOS | | 1.0 (0.7/0.3) | | 45 | 95 | 0 | 0.2 | 47.4±5.1 | | -17.3±1.5 | 7.32 | 2.93 | | 60 |
|  | TEOS | | 1.0 (0.7/0.3) | | 45 | 95 | 0.1 | 0.2 | 51.2±2.1 | | -23.2±1.1 | 5.48 | 2.26 | | 60 |
|  | TEOS | | 1.0 (0.7/0.3) | | 45 | 95 | 0.2 | 0.2 | 49.9±1.3 | | -23.2±1.1 | 3.15 | 1.46 | | 60 |
|  | TEOS | | 1.0 (0.7/0.3) | | 45 | 95 | 0.3 | 0.2 | 43.9±1.8 | | -17.5±2.6 | 0.99 | 0.52 | | 60 |

**Supplementary Table 2.** The comparison of specific surface area, pore volume, pore size with different MONs**.**

| TEOS (g) | BTES (g) | | | MPTES (g) | | Surface area  (m^2^ g^-1^) | Pore volume  (cm^3^ g^-1^) | | Pore size  （nm） | |  |
| --- | --- | --- | --- | --- | --- | --- | --- | --- | --- | --- | --- |
| 1.0 (0.7/0.3) | | 0 | 0.2 | | 1010.21 | | | 1.24 | | 2.3 | |
| 1.0 (0.7/0.3) | | 0.1 | 0.2 | | 841.99 | | | 1.56 | | 3.4 | |
| 1.0 (0.7/0.3) | | 0.2 | 0.2 | | 685.25 | | | 1.43 | | 3.3 | |
| 1.0 (0.7/0.3) | | 0.3 | 0.2 | | 668.86 | | | 1.24 | | 3.5 | |
| 1.0 (0.7/0.3) | | 0.1 | 0.15 | | 897.04 | | | 1.43 | | 2.5 | |
| 1.0 (0.7/0.3) | | 0.1 | 0.2 | | 841.99 | | | 1.56 | | 3.4 | |
| 1.0 (0.7/0.3) | | 0.1 | 0.3 | | 265.32 | | | 0.83 | | 2.1 | |

**Supplementary Table 3.** The comparison of thiol content, NO content and the release time of NO between present work and previously reported nanomaterials.

| Nanomaterials | Thiol content  (μmol mg^-1^) | | NO content  (µmol mg^-1^) | | Release time  ~ (h) | Reference |
| --- | --- | --- | --- | --- | --- | --- |
| MSN-SH | | 0.283 | N/A | N/A | | [^7^](#_ENREF_7) |
| MSN-SH | | 0.284 | N/A | N/A | | [^8^](#_ENREF_8) |
| MSN-SH | | 0.27 | N/A | N/A | | [^9^](#_ENREF_9) |
| MSN-SH | | 0.12 | N/A | N/A | | [^10^](#_ENREF_10) |
| MSN-SH | | 0.537 | N/A | N/A | | [^11^](#_ENREF_11) |
| MSN-SH | | N/A | 0.8 | 3 | | [^12^](#_ENREF_12) |
| Silica-SH | | N/A | 0.6 | 10 | | [^13^](#_ENREF_13) |
| Au–MSN-SH | | N/A | 0.14 | 0.5 | | [^14^](#_ENREF_14) |
| UCNPs@MSN-SH | | N/A | 0.22 | 10 | | [^15^](#_ENREF_15) |
| Fe_3_O_4_@PDA@MSN-SH | | N/A | 0.02 | 2 | | [^16^](#_ENREF_16) |
| HMTNPs-SH (TiO_2_) | | N/A | 1 | 0.1 | | [^17^](#_ENREF_17) |
| Cdot-TPP-SH | | N/A | 0.2 | 6 | | [^18^](#_ENREF_18) |
| PAMAM-SH | | N/A | 0.2 | 0.5 | | [^19^](#_ENREF_19) |
| PAA-SH | | N/A | 0.36 | 12 | | [^20^](#_ENREF_20) |
| Polypeptide-SH | | N/A | 0.2 | 1 | | [^21^](#_ENREF_21) |
| Chitosan-SH | | 1.1 | 0.3 | 0.1 | | [^22^](#_ENREF_22) |
| PAA-SH (polymer) | | N/A | 0.06 | 3 | | [^23^](#_ENREF_23) |
| SMA-tDod-SH | | N/A | 0.16 | 0.1 | | [^24^](#_ENREF_24) |
| MON-SH (dope) | | 3.65 | 2.26 | 48 | | This work |
| MON-SH (graft) | | 0.184 | 0.15 | 2 | | This work |

**Supplementary References**

1. J. Lu, F. Liu, H. Li, Y. Xu, S. Sun, Width-Consistent mesoporous silica nanorods with a precisely controlled aspect ratio for lysosome dysfunctional synergistic chemotherapy/photothermal therapy/starvation therapy/oxidative therapy. *ACS Applied Materials & Interfaces* **12** 24611-24622 (2020).

2. D. Shao, J. Li, X. Zheng, Y. Pan, Z. Wang, M. Zhang, Q. X. Chen, W. F. Dong, L. Chen, Janus "nano-bullets" for magnetic targeting liver cancer chemotherapy. *Biomaterials* **100** 118-133 (2016).

3. J. N. Chu, G. Traverso, Foundations of gastrointestinal-based drug delivery and future developments. *Nature Reviews Gastroenterology & Hepatology* **19** 219-238 (2022).

4. C. Shi, J. Dawulieti, F. Shi, C. Yang, Q. Qin, T. Shi, L. Wang, H. Hu, M. Sun, L. Ren, F. Chen, Y. Zhao, F. Liu, M. Li, L. Mu, D. Liu, D. Shao, K. W. Leong, J. She, A nanoparticulate dual scavenger for targeted therapy of inflammatory bowel disease, *Science Advances* **8** eabj2372 (2022).

5. P. Praveschotinunt, A. M. Duraj-Thatte, I. Gelfat, F. Bahl, D. B. Chou, N. S. Joshi, Engineered E. coli Nissle 1917 for the delivery of matrix-tethered therapeutic domains to the gut. *Nature Communications* **10** 5580 (2019).

6. T. E. Adolph, M. F. Tomczak, L. Niederreiter, H. J. Ko, J. Böck, E. Martinez-Naves, J. N. Glickman, M. Tschurtschenthaler, J. Hartwig, S. Hosomi, M. B. Flak, J. L. Cusick , K. Kohno, T. Iwawaki, S. Billmann-Born, T. Raine, R. Bharti, R. Lucius, M. N. Kweon, S. J Marciniak, A. Choi, S. J. Hagen, S. Schreiber, P. Rosenstiel, A. Kaser, R. S. Blumberg, Paneth cells as a site of origin for intestinal inflammation. *Nature* **503** 272-276 (2013).

7. M. Kalantari, Z. Gu, Y. Cao, C. Lei, J. Zhang, Thiolated silica nanoadsorbents enable ultrahigh and fast decontamination of mercury(ii): understanding the contribution of thiol moieties' density and accessibility on adsorption performance. *Environmental Science Nano* **7** 851-860 (2020).

8. M. Kalantari, T. Ghosh, Y. Liu, J. Zhang, J. Zou, C. Lei, C. Yu, Highly thiolated dendritic mesoporous silica nanoparticles with high-content gold as nanozymes: The nano-gold size matters. *ACS Applied Materials & Interfaces* **11**13264-13272 (2019).

9. A. Arencibia, M. S. Lopez-Gutierrez, J. M. Arsuaga, Efficient aqueous As(III) removal by adsorption on thiol-functionalized mesoporous silica. *Journal of Chemical Technology and Biotechnology* **95** 1883-1891 (2020).

10. X. Dong, H. J. Liu, H. Y. Feng, S. C. Yang, X. L. Liu, X. Lai, Q. Lu, J. F. Lovell, H. Z. Chen, C. Fang, Enhanced drug delivery by nanoscale integration of a nitric oxide donor to induce tumor collagen depletion. *Nano Letters* **19** 997-1008 (2019).

11. M. J. Malone-Povolny, M. H. Schoenfisch, Extended nitric oxide-rleasing polyurethanes via S-nitrosothiol-modified mesoporous silica nanoparticles. *ACS Applied Materials & Interfaces* **11** 12216-12223 (2019).

12. M. H. Kafshgari, A. Cavallaro, B. Delalat, F. J. Harding, S. J. McInnes, E. Mäkilä, J. Salonen, K. Vasilev, N. H. Voelcker, Nitric oxide-releasing porous silicon nanoparticles. *Nanoscale Research Letters* **9** 333 (2014).

13. D. L. Slomberg, Y. Lu, A. D. Broadnax, R. A. Hunter, A. W. Carpenter, M. H. Schoenfisch, Role of size and shape on biofilm eradication for nitric oxide-releasing silica nanoparticles. *ACS Applied Materials & Interfaces* **5** 9322-9329 (2013).

14. P. Liu, Y. Wang, Y. Liu, F. Tan, J. Li, N. Li, S-nitrosothiols loaded mini-sized Au@silica nanorod elicits collagen depletion and mitochondrial damage in solid tumor treatment. *Theranostics* **10** 6774-6789 (2020).

15. W. Fan, W. Bu, Z. Zhang, B. Shen, H. Zhang, Q. He, D. Ni, Z. Cui, K. Zhao, J. Bu, J. Du, J. Liu, J. Shi, X-ray radiation-controlled NO-release for on-demand depth-independent hypoxic radiosensitization. *Angewandte Chemie-International Edition* **54** 14026-14030 (2015).

16. R. Guo, Y. Tian, Y. Wang, W. Yang, Near-Infrared Laser-Triggered nitric oxide nanogenerators for the reversal of multidrug resistance in cancer. *Advanced Functional Materials* **27** 1606398 (2017).

17. Q. Feng, Y. Li, X. Yang, W. Zhang, Y. Hao, H. Zhang, L. Hou, Z. Zhang, Hypoxia-specific therapeutic agents delivery nanotheranostics: A sequential strategy for ultrasound mediated on-demand tritherapies and imaging of cancer. *Journal of Controlled Release* **275** 192-200 (2018).

18. J. Xu, F. Zeng, H. Wu, C. Hu, C. Yu, S. Wu, Preparation of a mitochondria-targeted and NO-Releasing nanoplatform and its enhanced pro-apoptotic effect on cancer cells. *Small* **10** 3750-3760 (2014).

19. K. Wang, M. Jiang, J. Zhou, Y. Liu, Q. Zong, Y. Yuan, Tumor-acidity and bioorthogonal chemistry-mediated on-site size transformation clustered nanosystem to overcome hypoxic resistance and enhance chemoimmunotherapy. *ACS Nano* **16** 721-735 (2022).

20. H. Jeong, D. Choi, Y. Oh, J. Heo, J. Hong, A nanocoating co-localizing nitric oxide and growth factor onto individual endothelial cells reveals synergistic effects on angiogenesis. *Advanced Healthcare Materials* **11** e2102095 (2022).

21. Y. Ding, C. Du, J. Qian, C. M. Dong, NIR-Responsive polypeptide nanocomposite generates NO gas, mild photothermia, and chemotherapy to reverse multidrug-resistant cancer. *Nano Letters* **19** 4362-4370 (2019).

22. Y. Lu, A. Shah, R. A. Hunter, R. J. Soto, M. H. Schoenfisch, S-Nitrosothiol-modified nitric oxide-releasing chitosan oligosaccharides as antibacterial agents. *Acta Biomaterialia* **12**, 62-69 (2015).

23. Z. Yang, D. Gao, X. Guo, L. Jin, J. Zheng, Y. Wang, S. Chen, X. Zheng, L. Zeng, M. Guo, X. Zhang, Z. Tian, Fighting immune cold and reprogramming immunosuppressive tumor microenvironment with red blood cell membrane-camouflaged nanobullets. *ACS Nano* **14** 17442-17457 (2020).

24. H. Alimoradi, A. Barzegar-Fallah, I. A. Sammut, K. Greish, G. I. Giles, Encapsulation of tDodSNO generates a photoactivated nitric oxide releasing nanoparticle for localized control of vasodilation and vascular hyperpermeability. *Free Radical Biology and Medicine* **130** 297-305 (2019).
